# Behavioral origin of sound-evoked activity in mouse visual cortex

**DOI:** 10.1038/s41593-022-01227-x

**Published:** 2023-01-09

**Authors:** Célian Bimbard, Timothy P. H. Sit, Anna Lebedeva, Charu B. Reddy, Kenneth D. Harris, Matteo Carandini

**Affiliations:** 1 UCL Institute of Ophthalmology, University College London, London, UK; 2 Sainsbury Wellcome Centre, University College London, London, UK; 3 UCL Queen Square Institute of Neurology, University College London, London, UK

**Keywords:** Sensory processing, Striate cortex, Cortex

## Abstract

Sensory cortices can be affected by stimuli of multiple modalities and are thus increasingly thought to be multisensory. For instance, primary visual cortex (V1) is influenced not only by images but also by sounds. Here we show that the activity evoked by sounds in V1, measured with Neuropixels probes, is stereotyped across neurons and even across mice. It is independent of projections from auditory cortex and resembles activity evoked in the hippocampal formation, which receives little direct auditory input. Its low-dimensional nature starkly contrasts the high-dimensional code that V1 uses to represent images. Furthermore, this sound-evoked activity can be precisely predicted by small body movements that are elicited by each sound and are stereotyped across trials and mice. Thus, neural activity that is apparently multisensory may simply arise from low-dimensional signals associated with internal state and behavior.

## Abstract (web summary)

Sounds evoke activity in visual cortex. Bimbard et al. find that this activity is stereotyped across cells, not specific to visual cortex, independent of inputs from auditory cortex and predicted by stereotyped movements elicited by the sounds.

## Main

Many studies suggest that all cortical sensory areas, including primary ones, are multisensory^1^. For instance, mouse’s primary visual cortex (V1) is influenced by sounds. Sounds may provide V1 with global inhibition^2^, modify the neurons’ tuning^3,4^, boost detection of visual events^5^ or even provide tone-specific information, reinforced by prolonged exposure^6^ or training^7^. This sound-evoked activity is thought to originate from direct projections from the auditory cortex^2,3,5,7^—it may be suppressed by inhibition of the auditory cortex^2,5^, and it may be mimicked by stimulation of auditory fibers^2,3^.

Here we consider a possible alternative explanation for these multisensory signals, based on low-dimensional changes in internal state and behavior^8,9^. Behavioral and state signals have profound effects on sensory areas. For instance, the activity of V1 neurons carries strong signals related to running^10,11^, pupil dilation^11,12^, whisking^13^ and other movements^14^. These behavioral and state signals are low-dimensional and largely orthogonal^13^ to the high-dimensional code that V1 uses to represent images^15^.

We hypothesized, therefore, that the activity evoked by sounds in V1 reflects sound-elicited changes in internal state and behavior. This seems possible because sounds can change internal state and evoke uninstructed body movements^16–20^. This hypothesis predicts that sound-evoked activity in V1 should have the typical attributes of behavioral signals: low dimension^13^ and a broad footprint^14,21,22^ that extends beyond the cortex^13^. Moreover, sound-evoked activity should be independent of direct inputs from auditory cortex and should be predictable from the behavioral effects of sounds.

To test these predictions, we recorded the responses of hundreds of neurons in mouse V1 to audiovisual stimuli, while filming the mouse to assess the movements elicited by the sounds. As predicted by our hypothesis, the activity evoked by sounds in V1 had a low dimension: it was largely one-dimensional. Moreover, it was essentially identical to activity evoked in another brain region, the hippocampal formation. Furthermore, it was independent of direct projections from auditory cortex, and it tightly correlated with the uninstructed movements evoked by the sounds. These movements were small but specific to each sound and stereotyped across trials and across mice. Thus much of the multisensory activity that has been observed in visual cortex may have a simpler, behavioral origin.

## Results

To explore the influence of sounds on V1 activity, we implanted Neuropixels 1.0 and 2.0 probes^23,24^ in eight mice and recorded from them during head fixation while playing naturalistic audiovisual stimuli. We selected 11 naturalistic movie clips^25^, each made of a video (gray-scaled) and a sound (loudness, 50–80 dB SPL; Supplementary Fig. 1), together with a blank movie (gray screen, no sound). On each trial, we presented a combination of the sound from one clip and the video from another (144 combinations repeated four times, in random order). Most neurons were recorded from layers 4 to 6.

### Sounds evoke stereotyped responses in visual cortex

**Fig. 1:**
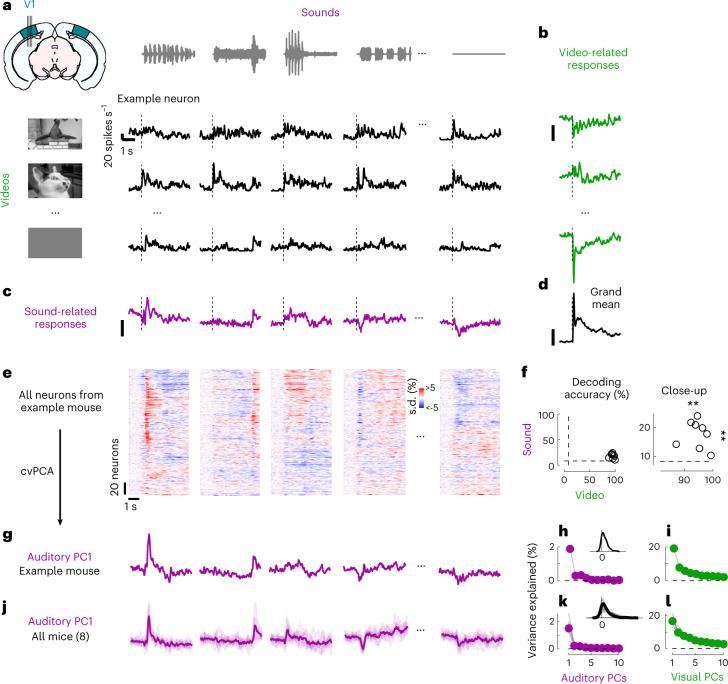
Sounds evoke stereotyped responses in visual cortex. **a**, Responses of an example neuron to combinations of sounds (columns) and videos (rows). Responses were averaged over four repeats. **b**, Video-related time courses (averaged over all sound conditions, minus the grand average) for the example neuron in **a**. **c**, Same, for the sound-related time courses. **d**, Grand average over all conditions for the neuron. Scale bars in **b**–**d**: 20 spikes per second. **e**, Sound-related time courses for all 212 neurons in one experiment, sorted using rastermap^13^. **f**, Decoding accuracy for video versus sound (double asterisks indicate *P* = 0.0039, Wilcoxon right-tailed signed rank test, *n* = 8 mice). Dashed lines show chance level (1/12). **g**, Time courses of the first principal component of the sound-related responses in **e** (‘auditory PC1’, arbitrary units). **h**, Fraction of total variance explained by auditory PCs, for this example mouse; inset: distribution of the weights of auditory PC1 (arbitrary units), showing that weights were typically positive. **i**, Same, for visual PCs. **j**–**l**, Same as **g**–**i**, for individual mice (thin curves) and averaged across mice (thick curves).

We then identified the visual and auditory components of each neuron’s sensory response. A typical V1 neuron responded differently to different combinations of videos and sounds (Fig. 1a). To characterize these responses, we used a marginalization procedure similar to factorial ANOVA. To measure the neuron’s video-related responses (Fig. 1b), we computed its mean response to each video, averaged across all concurrent sounds. Similarly, to characterize the neuron’s sound-related responses (Fig. 1c), we computed the mean response to each sound, averaged across all concurrent videos. These measures were then ‘marginalized’ by subtracting the grand average over all videos and sounds (Fig. 1d).

Sounds evoked activity in a large fraction of V1 neurons, and this activity was reliably different across sounds. Some sounds barely evoked any activity, while others evoked stereotyped responses, at different points in time (Fig. 1e). From the marginalized single-trial population responses, we could decode not only the identity of each video (with 95 ± 1% accuracy, s.e., *P* = 0.0039, Wilcoxon right-tailed signed rank test, *n* = 8 mice) but also the identity of each sound (with 18 ± 2% accuracy, *P* = 0.0039, Wilcoxon right-tailed signed rank test, *n* = 8 mice; Fig. 1f).

The activity evoked by sounds was so stereotyped across responsive neurons that it was essentially one-dimensional. We analyzed the sound-related population responses with cross-validated Principal Component Analysis^15^ (cvPCA) and found that the time course of the first principal component (PC) of sound-related responses (‘auditory PC1’) for each sound was similar to the responses evoked in individual neurons (Fig. 1g). This first dimension explained most (55%) of the cross-validated sound-related variance (1.9% of the total variance) with subsequent dimensions explaining much smaller fractions (Fig. 1h). Furthermore, neurons showed distributed yet overall positive weights on this first PC, indicating a largely excitatory effect of sound (Fig. 1h, inset).

**Extended Data Fig. 1:**
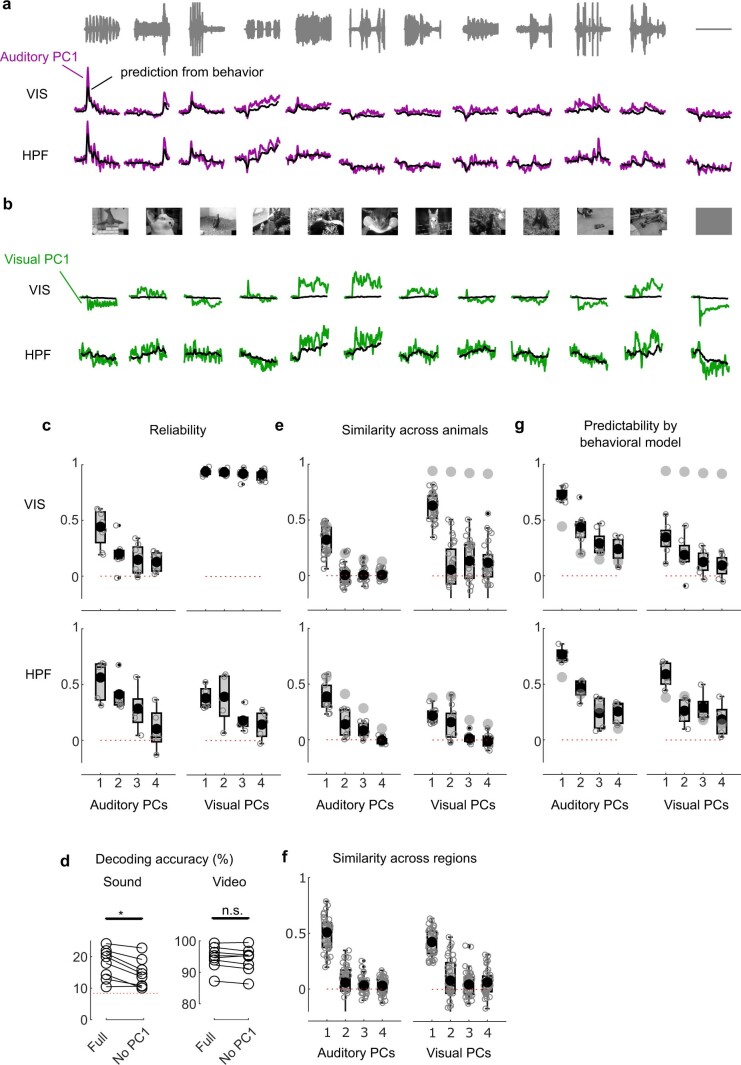
Coding of visual vs. auditory stimuli in visual cortex and hippocampal formation. **a**. Time courses of the auditory PC1 averaged across mice (z-scored), measured in visual cortex (VIS, *top*) and hippocampal formation (HPF, *bottom*). Traces show the actual data (purple) and the cross-validated prediction from the behavioral model (black). **b**. Same as **a**, but for visual PC1 (green). **c**. Reliability of each auditory (*left*) or visual (*right*) PC, in VIS (*top*, n = 8 mice) or HPF (*bottom*, n = 5 mice). The large dot shows the z-transformed mean; the bounds of each box show the 25^th^ and 75^th^ percentiles; the whiskers show the minimum and maximum values that are not outliers; small dots show outliers (computed using the interquartile range); individual dots are also shown. **d**. Decoding accuracy of sound identity from auditory PCs (*left*) or video identity from visual PCs (*right*) measured in VIS, taking the full subspace or the full subspace except PC1. Sound decoding was significantly worse without auditory PC1 (*: p = 0.0156, two-sided paired Wilcoxon sign rank, n = 8 mice). **e**. Same as **c** but showing the similarity across animals. Reliability of each PC is shown for reference (gray, replotted from **c**). **f**. Similarity of visual and auditory PCs between VIS and HPF. **g**. Same as **e**, for the predictability of each PC by the behavioral model, measured by the cross-validated correlation between data and model prediction. The model can sometimes predict the test set better than the train set because it can predict fluctuations specific to the test set.

Similar results held across mice: the activity evoked by sounds in V1 was largely one-dimensional (auditory PC1 explained 53 ± 3% of the sound-related variance, s.e., *n* = 8 mice), and the first principal component across mice had similar time courses and similar dependence on sound identity (Fig. 1j,k). Indeed, the correlation of auditory PC1 time courses evoked in different mice was 0.34, close to the test–retest correlation of 0.44 measured within individual mice (Extended Data Fig. 1c,e). Again, in all mice, the neuron’s weights for the auditory PC1 were widely distributed, with a positive bias (*P* = 0.0078, Wilcoxon two-tailed signed rank test on the mean, *n* = 8 mice; Fig. 1k). Higher-order PCs were harder to compare across mice (Extended Data Fig. 1c,e). Components 2, 3 and 4 also encoded auditory stimuli markedly but explained much less variance (Fig. 1h and Extended Data Fig.1c,d). Thus sounds evoke essentially one-dimensional population activity, which follows a similar time course even across brains. In the rest of the paper, we will illustrate sound-evoked activity by using the time course of this single ‘auditory PC1’.

In contrast, the activity evoked by videos in V1 neurons was markedly larger and higher dimensional. The first visual PC explained a much higher fraction of total variance than the first auditory PC (17 ± 1% versus 1.5 ± 0.3% s.e., *n* = 8 mice; Fig. 1i,l). Furthermore, higher visual PCs explained substantial amounts of variance^15^.

### Sounds evoke stereotyped responses in hippocampal formation

**Fig. 2:**
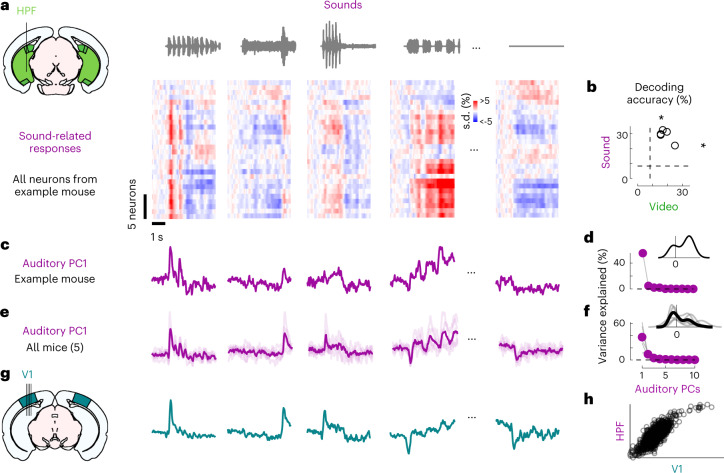
Sounds evoke stereotyped responses in hippocampal formation. **a**, Sound-related time courses for all 28 neurons in HPF in one experiment, sorted using rastermap^13^. **b**, Decoding accuracy for video versus sound (asterisk indicates *P* = 0.031, Wilcoxon right-tailed signed rank test, *n* = 5 mice). Dashed lines show chance level (1/12). **c**, Time courses of the first principal component of the sound-related responses in **a** (‘auditory PC1’, arbitrary units). **d**, Fraction of total variance explained by auditory PCs, for this example mouse; inset: distribution of the weights of auditory PC1 (arbitrary units). **e**,**f**, Same as **c** and **d** for individual mice (thin curves) and average of all mice (thick curves). **g**, Time courses of the auditory PC1 in visual cortex (from Fig. 1), for comparison. **h**, Comparison of the auditory PC1 from HPF (from **e**) and from V1 (from Fig. 1); arbitrary units.

We next investigated whether these auditory-evoked signals were specific to visual cortex. Thanks to the length of Neuropixels probes, while recording from V1, we simultaneously recorded from the hippocampal formation (dorsal subiculum and prosubiculum, dentate gyrus and CA3; Fig. 2). These regions receive little input from auditory cortex and auditory thalamus^26^.

**Extended Data Fig. 2:**
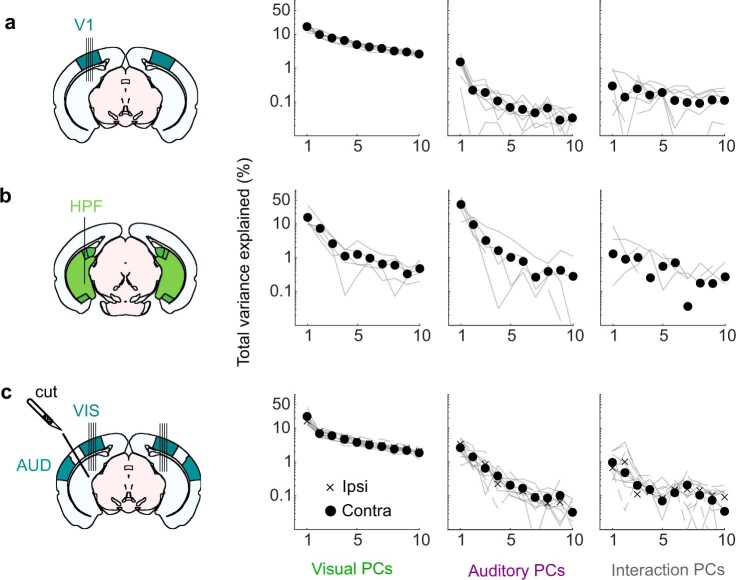
Dimensionality of auditory and visual responses in visual cortex and hippocampal formation. **a**. Top: Total variance explained (normalized test-retest covariance) for visual PCs (*left*), auditory PCs (*middle*) and interactions PCs (*right*), for all 8 recordings in V1 (thin lines) and their average (filled dots). The total variance is measured from the normalized test-retest covariance, which can occasionally be negative (not visible in logarithmic scale). **b**. Same as **a** but with the 5 recordings from the HPF. **c**. Same as **a** but with the 12 recordings from the visual cortices ipsilateral (6, crosses) and contralateral (6, filled dots) to the cut (6 sessions across 3 mice).

Sounds evoked strong activity in the hippocampal formation (HPF), and this activity was largely similar across cells and different across sounds (Fig. 2a). As in visual cortex, the activity in single trials could be used to decode sound identity (29 ± 2% and to a lesser extent video identity 19 ± 2%, *P* = 0.031 for both, Wilcoxon right-tailed signed rank test, *n* = 5 mice; Fig. 2b). Projection of the sound-related activity along the auditory PC1 showed different time courses across sounds (Fig. 2c,e), and this first PC explained most of the sound-related variance (65 ± 13% Fig. 2d,f). Similarly, the representation of videos was also low dimensional (Extended Data Fig. 2b).

The activity evoked by sounds in the hippocampal formation was remarkably similar to the activity evoked in visual cortex. Indeed, the time courses of the auditory PC1 in the two regions, averaged over mice, were hardly distinguishable (compare Fig. 2e–g and see Extended Data Fig. 1a,f), with a correlation of *r* = 0.82 (Fig. 2h). Because they explain much less variance, higher-order PCs were more variable across regions (Extended Data Fig. 1f). The time course of the visual PC1 also shared similarities with the visual PC1 found in visual cortex, but higher-order PCs did not (Extended Data Fig. 1b,f).

### Sound responses are not due to inputs from auditory cortex

**Fig. 3:**
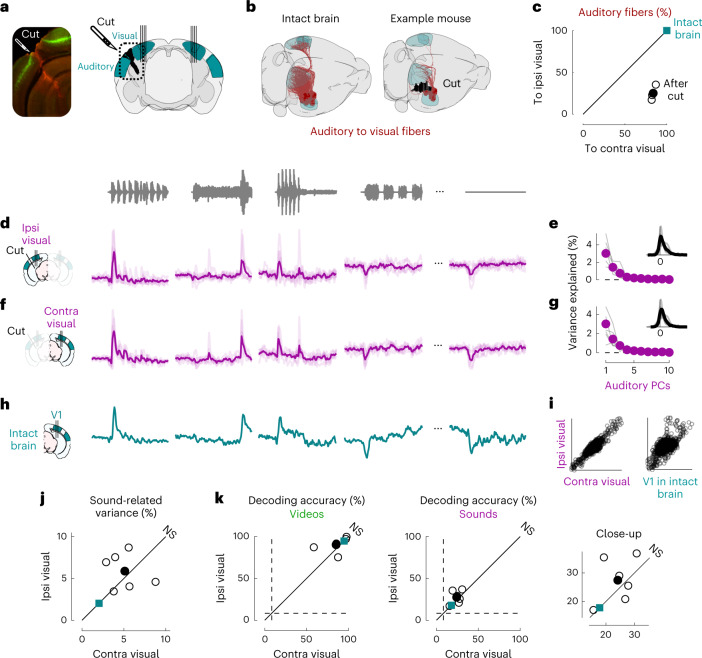
Sound responses in visual cortex are not due to inputs from auditory cortex. **a**, Coronal views of a transectomy cutting the connections between auditory and visual cortex in one hemisphere, showing histology (left) and reconstruction of the cut (right). After the cut, bilateral recordings are performed in visual cortex. **b**, Three-dimensional visualizations showing auditory to visual fibers (red) in an intact brain (top) versus after the cut (bottom) in an example mouse. **c**, Auditory input to the sides contralateral versus ipsilateral to the cut for all three mice (open dots) and their average (filled dot), normalized by the input expected in intact brains (turquoise dot). **d**, Time courses of the first principal component of the sound-related responses (‘auditory PC1’) on the side ipsilateral to the cut (average over all mice). Thin curved lines show individual mice. **e**, Fraction of total variance explained by auditory PCs on the side ipsilateral to the cut; inset: distribution of the weights of auditory PC1 for all mice. **f**,**g**, Same as **d** and **e** for the side contralateral to the cut. **h**, Time courses of the auditory PC1 in visual cortex of intact, control mice (from Fig. 1) for comparison. **i**, Comparison of the auditory PC1 from the sides contralateral and ipsilateral to the cut (left, from **d** versus **f**) and from V1 (right, taken from **b** versus Fig. 1); all arbitrary units. **j**, Sound-related variance explained by the first four auditory PCs on the ipsi- versus contralateral side, showing individual sessions (open dots), their average (black dot) and the average across control mice (turquoise dot) (Wilcoxon two-sided paired signed rank test, *n* = 6 sessions across three mice). **k**, Decoding accuracy for videos (left) and sounds (middle and right, showing close-up) (Wilcoxon two-sided paired signed rank test, *n* = 6 sessions across three mice). Symbols are as in **j**.

**Extended Data Fig. 3:**
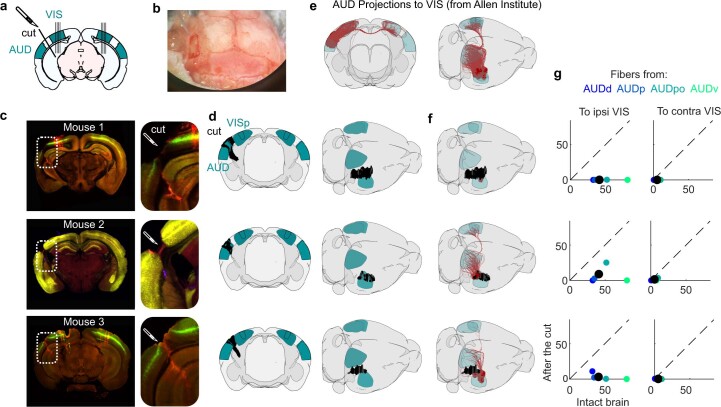
Transectomy cut most of the fibers from auditory to visual cortex. **a**. Schematic of the transectomy experiments: the connections between auditory (AUD) and visual (VIS) cortex are cut on one side. Subsequently, recordings are performed in visual cortex, in both hemispheres. **b**. Picture from above of the mouse skull during surgery, with a craniotomy performed on the left side. **c**. Histology of the three mouse brains, showing the cut (*inset*), and the probe tracks (DiI and DiO staining, mainly visible in mice 1 and 3). **d**. 3D reconstructions of the cut, shown from a coronal view (*left*) or from above/sideways (*right*). **e**. Fiber tracks from the auditory cortex to the visual cortex in intact mice, from 53 experiments performed in the Allen Mouse Brain Connectivity atlas^26^, see Methods). **f**. Estimated intact fibers after the cut, for the 3 mice. **g**. Estimate of the number of fibers before the cut (abscissa) and after the cut (ordinate) for each mouse, in ipsilateral (*left*) and contralateral visual cortex (*right*). The color of the dots indicates the auditory area from which the fibers originated (Allen Mouse Brain Connectivity Atlas). The black dot shows the average over all 53 experiments performed for the Atlas.

We next returned to the activity evoked by sounds in visual cortex and asked if this activity is due to projections from auditory cortex, as has been proposed^2,3,5,7^. We performed transectomies^2^ to cut the fibers between auditory and visual areas in one hemisphere and recorded bilaterally while presenting our audiovisual stimuli (Fig. 3a). The cut ran along the whole boundary between auditory and visual areas and was deep enough to reach into the white matter (Extended Data Fig. 3a–c). We carefully quantified the precise location and extent of the cut in 3D, based on the histology (Fig. 3b and Extended Data Fig. 3d). To estimate the fraction of fibers from auditory to visual areas that were cut, we extracted the trajectories of those fibers from the Allen Mouse Brain Connectivity Atlas^26^ and intersected them with the location of our cut. We thus estimated that the cut decreased the total input from the two auditory cortices to the visual areas ipsilateral to the cut by an average factor of >3.6 compared to the contralateral side (4.8, 2.5 and 3.6 in the three mice; Fig. 3c and Extended Data Fig. 3e–g). Thus, if auditory-evoked activity in visual cortex originates from auditory cortex, it should be drastically reduced on the cut side.

The activity evoked by sounds in visual cortex was similar on the cut and the uncut side. Indeed, the time course of the activity projected along auditory PC1 on the side of the cut (Fig. 3d,e) was essentially identical to the time course of auditory PC1 in the opposite hemisphere (*r* = 0.9, Fig. 3f,g) and barely distinguishable from the one measured in the control mice (cut, *r* = 0.62 and uncut, *r* = 0.56; Fig. 3h,i). Their relative timing was also identical, with a cross-correlation (measured at 1-ms resolution) that peaked at 0 delays. The distribution of the variance explained by the first auditory PCs and the distribution of neuronal weights on the auditory PC1 were similar on the two sides (Fig. 3e versus g). The total variance of the activity related to sounds on the cut side was on average equal to the sound-related variance on the uncut side (Fig. 3j and see Extended Data Fig. 2c for all eigenspectra) and was significantly larger than expected from the few auditory fibers that were spared by the transectomies (*P* = 0.031, Wilcoxon two-tailed paired signed rank test, *n* = 6 experiments across three mice). Furthermore, decoding accuracy was similar across sides for both sounds (cut, 27 ± 3% and uncut, 24 ± 2%; *P* = 0.016 for both, Wilcoxon right-tailed signed rank test; comparison: *P* = 0.44, Wilcoxon two-sided paired signed rank test) and videos (cut, 90 ± 4% and uncut, 85 ± 6%; *P* = 0.016 for both, Wilcoxon right-tailed signed rank test; comparison: *P* = 0.31, Wilcoxon two-sided paired signed rank test; Fig. 3k).

These results indicate that the activity evoked by sounds in visual cortex in our experiments cannot be explained by direct inputs from auditory cortex.

### Sounds evoke stereotyped uninstructed behaviors

**Fig. 4:**
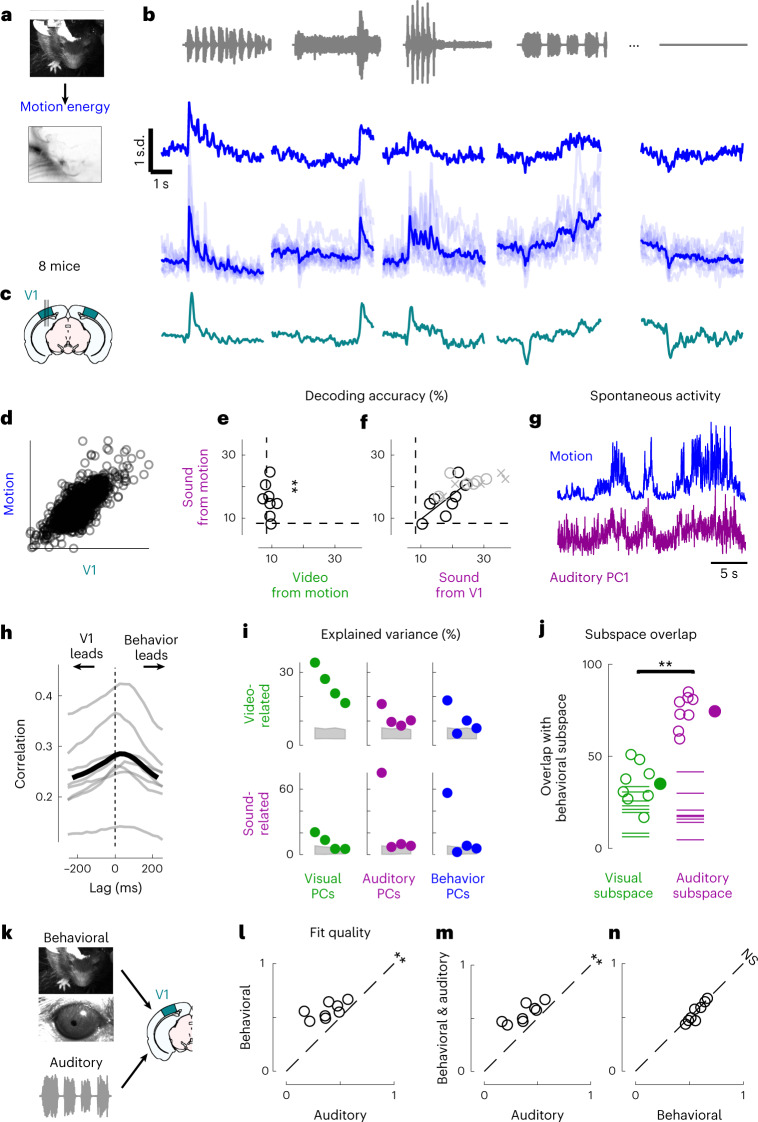
Sounds evoke stereotyped, uninstructed behaviors that predict sound responses in visual cortex. **a**, Extraction of motion PCs from videos of the mouse face. **b**, Sounds evoked changes in the first motion PC, both in an example mouse (top) and all mice (bottom). Scale bar: 1 s.d. **c**, Time courses of the auditory PC1 in visual cortex (from Fig. 1). **d**, Comparison of the time courses of motion (taken from **b**) and of the auditory PC1 from V1 (taken from Fig. 1); all arbitrary units. **e**, Decoding of sound identity from the first 128 motion PCs was significantly above chance level (dashed lines) (double asterisks indicate *P* = 0.0078, Wilcoxon right-tailed signed rank test, *n* = 8 mice). **f**, Across mice, there was a strong correlation between the accuracy of sound decoding from facial motion and from V1 activity. The linear regression is performed on the control mice from Fig. 1 (black dots). Data from transectomy mice (gray markers) confirm the trend, both on the cut side (crosses) and on the uncut side (circles). **g**, Time course of facial motion (top) and of V1 activity along auditory PC1 (bottom) in the absence of any stimulus, for an example mouse. **h**, Cross-correlogram of these time courses, for individual mice (gray) and their average (black). The positive lag indicates that movement precedes neural activity. **i**, Video- and sound-related variance explained by neural activity along the visual (left), auditory (middle) or behavioral (right) subspaces (first four PCs of each subspace), for one example mouse. The gray regions show 90% confidence intervals expected by chance (random components). **j**, Overlap between the auditory or the visual subspace and the behavioral subspace for each mouse (open dots) and all mice (filled dot) (double asterisks indicate *P* = 0.0078, Wilcoxon two-sided paired signed rank test, *n* = 8 mice). Dashed lines show the significance threshold (95th percentile of the overlap with random dimensions) for each mouse. **k**, Schematics of the three encoding models trained to predict the average sound-related activity in the auditory subspace. **l**–**n**, Cross-validated correlation of the actual sound responses and their predictions for all mice, comparing different models (auditory, behavioral and full; double asterisks indicate *P* = 0.0078, Wilcoxon two-sided paired signed rank test, *n* = 8 mice).

**Extended Data Fig. 4:**
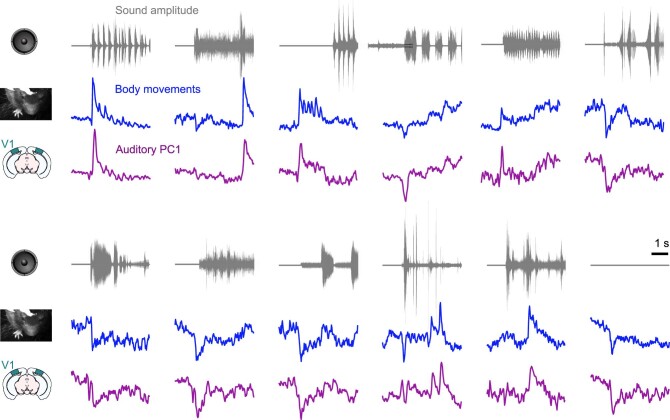
Neural and behavioral responses differ across sounds but resemble each other. Responses along neural auditory PC1 from V1 (*purple*), and motion energy (*blue*) for all sounds. Responses are averaged over repeats, videos, and mice, and z-scored. The top trace (*gray*) shows the envelope of the corresponding sound. As in all main text figures, these responses are expressed relative to the grand average over sounds and videos; this explains the negative deflections seen in the responses to the blank stimulus.

**Extended Data Fig. 5:**
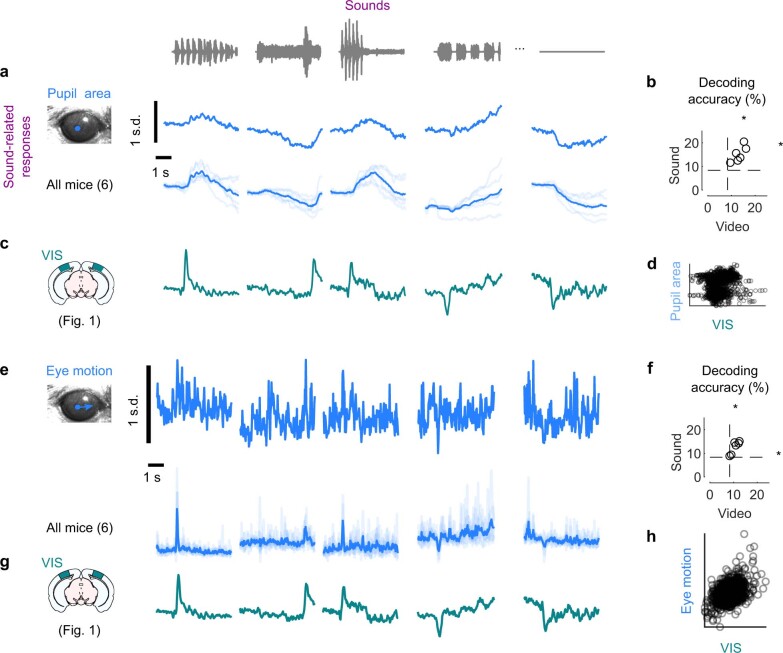
Sounds trigger changes in arousal and eye movements. **a**. Sound-related responses changes in arousal as measured by pupil diameter, for one example mice, and all mice (6 of 8 were monitored with an eye camera). **b**. Decoding of video and sound identity using pupil area. (*: p = 0.0156, right-tailed Wilcoxon sign rank test, n = 6 mice) **c**. Comparison with the time course of the neural auditory PC1 from Fig. 1. **d**. Scatter plot of auditory PC1 vs. pupil area. **f-h**. Same as **a-d**. but for eye movements (in f, *: p = 0.0312 for videos and 0.0156 for sounds).

Sounds evoked uninstructed body movements that were small but stereotyped across trials and mice and different across sounds. To measure body movements, we used a wide-angle camera that imaged the head, front paws and back (Fig. 4a). Sounds evoked a variety of uninstructed movements, ranging from rapid startle-like responses <50 ms after sound onset to more complex, gradual movements (Fig. 4b and see Extended Data Fig. 4 for all sounds). These movements were remarkably similar across trials and mice. The main and most common type of sound-evoked movements were subtle whisker twitches (Supplementary Video 1), which we quantified by plotting the first principal component of facial motion energy^13^ (Fig. 4b). These movements were influenced by sound loudness, and to some extent by frequency, but not by spatial location (Supplementary Fig. 2). Moreover, sounds evoked stereotyped changes in arousal, as observed by the time courses of pupil size, which were highly consistent across trials and mice (Extended Data Fig. 5).

Because sound-evoked movements were different across sounds and similar across trials, we could use them to decode sound identity with 16 ± 2% accuracy (s.e., *P* = 0.0078, Wilcoxon right-tailed signed rank test, *n* = 8 mice; Fig. 4e). This accuracy was not significantly different from the 18 ± 2% accuracy of sound decoding from neural activity in visual cortex (*P* = 0.15, Wilcoxon two-sided paired signed rank test), suggesting a similar level of single-trial reliability in behavior and in neural activity.

### Behavior predicts sound-evoked responses in visual cortex

The body movements evoked by sounds had a remarkably similar time course to the activity evoked by sounds in area V1 (Fig. 4b,c). The two were highly correlated across time and sounds (*r* = 0.75; Fig. 4d and Extended Data Fig. 4). Furthermore, the accuracy of decoding sound identity from V1 activity and from behavior was highly correlated across mice (*r* = 0.73, *P* = 0.041, F-statistic versus constant model, *n* = 8 mice; Fig. 4f), suggesting that sound-specific neural activity was higher in mice that moved more consistently in response to sounds. As it happens, the cohort of transectomy mice showed higher sound decoding accuracy from their behavior compared to the main cohort. Consistent with our hypothesis, these same mice showed higher sound decoding accuracy from their V1 activity, regardless of hemisphere.

**Extended Data Fig. 6:**
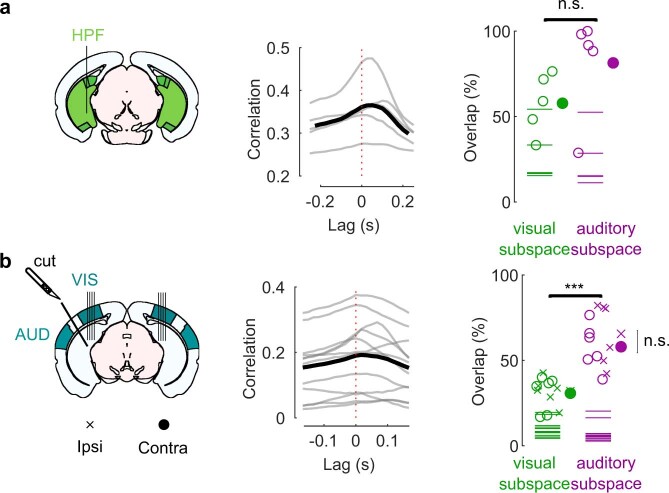
Timing of movements and sound-related neural activity and overlap between neural subspaces related to behavior and sounds. **a**. *Left***:** Cross-correlogram of the motion energy and the neural activity on the auditory PC1 during the spontaneous period, for individual mice (*gray*) and averaged across mice (*black*). A positive lag means that movement preceded neural activity. *Right*: Overlap between the neural subspace related to behavior and the subspace related to video (*left*) or to sound (*right*), for each mouse (*open dots*) and averaged across mice (*filled dot*). Dashed lines show the significance threshold (95^th^ percentile of the overlap with random dimensions) for each mouse (two-sided paired Wilcoxon sign rank test, n = 5 mice). **b**. Same as **a** for the recordings in visual cortex after a transectomy (***: p = 0.00048, two-sided paired Wilcoxon sign rank test, n = 12 recordings across 3 mice; comparison cut vs. uncut side: two-sided paired Wilcoxon sign rank test, n = 6 sessions across 3 mice).

Moreover, the neural activity along auditory PC1 correlated with movements even during spontaneous behavior, when no stimulus was presented (*r* = 0.29 ± 0.03, s.e.; Fig. 4g,h). Movement preceded neural activity by a few tens of milliseconds (28 ± 7 ms, s.e., *P* = 0.031, Wilcoxon two-sided signed rank test, *n* = 8 mice; Fig. 4h and see Extended Data Fig. 6 for the hippocampal formation and for both sides of the visual cortex in transectomy experiments).

Another similarity between the neural activities evoked by sounds and by movement could be seen in their subspaces^13^, which substantially overlapped with each other. To define the behavioral subspace, we applied reduced-rank regression to predict neural activity from movements during the spontaneous period (in the absence of stimuli). This behavioral subspace largely overlapped with the auditory subspace: the first four components of the movement-related subspace explained 75 ± 3% (s.e.; *P* < 0.05 for all mice separately, randomization test) of the sound-related variance, much more than the video-related variance^13^ (35 ± 4%, comparison: *P* = 0.0078, Wilcoxon two-sided paired signed rank test; Fig. 4i,j). We observed a similar overlap in the hippocampal formation, and on both sides of visual cortex in the transectomy experiments (Extended Data Fig. 6).

We then asked to what extent body movements could predict sound-evoked neural activity in V1. We fitted three models to the sound-related single-trial responses (projected onto the full auditory subspace) and used the models to predict trial averages of these sound responses on a different test set (Fig. 4k and Supplementary Fig. 3). The first was a purely auditory model where the time course of neural activity depends only on sound identity. This model is equivalent to a test–retest prediction, so it will perform well regardless of the origin of sound-evoked activity; it would fit perfectly with an infinite number of trials. The second was a purely behavioral model where neural activity is predicted by pupil area, eye position and motion, and facial movements. This model would perform well only if behavioral variables observed in individual trials do predict the trial-averaged sound-evoked responses. The third was a full model where activity is due to the sum of both factors, auditory and behavioral.

**Extended Data Fig. 7:**
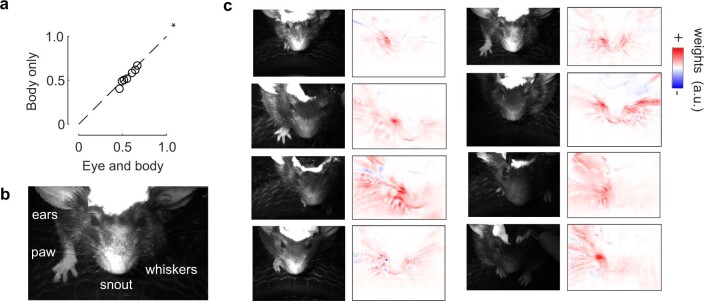
Sound-evoked V1 responses are mainly explained by whisker movements. **a**. Correlation of the actual data and their predictions for all mice, comparing a model containing both eye and body movements predictors (‘Eye and body’) to a model containing only body movements predictors (‘Body only’). The eye predictors only marginally increase the fit prediction accuracy (*: p = 0.039, two-sided paired Wilcoxon sign rank test, n = 6 mice), suggesting that body movements are the best and main predictors. **b**. Example frame of the face, with the parts of the body that were visible. **c**. For each mouse, we analyzed the image of the mouse (*left*) and obtained the weights that best predicted the auditory PC1 (*right*). Most of the weights are related to the whiskers. The asymmetry of the weight distribution across the two sides of the face is likely due to differences in lighting.

This analysis revealed that the sounds themselves were unnecessary to predict sound-evoked activity in visual cortex, whereas the body movements elicited by sounds were sufficient. As expected, the auditory model was able to capture much of this activity. However, it performed worse than the full model and the behavioral model (*P* = 0.0078, Wilcoxon two-sided paired signed rank test; Fig. 4l,m). These models captured not only the average responses to the sounds (see Extended Data Fig. 1a for time courses across all sounds), but also the fine differences in neural activity between the train and test set, which the auditory model cannot predict (because the two sets share the same sounds). Remarkably, the behavioral model performed just as well as the full model (*P* = 0.25, Wilcoxon two-sided paired signed rank test; Fig. 4n), indicating that the sounds themselves were unnecessary to predict sound-evoked activity. Further analysis indicated that the main behavioral correlates of sound-evoked activity in V1 were movements of the body and of the whiskers, rather than the eyes (Extended Data Fig. 7).

**Extended Data Fig. 8:**
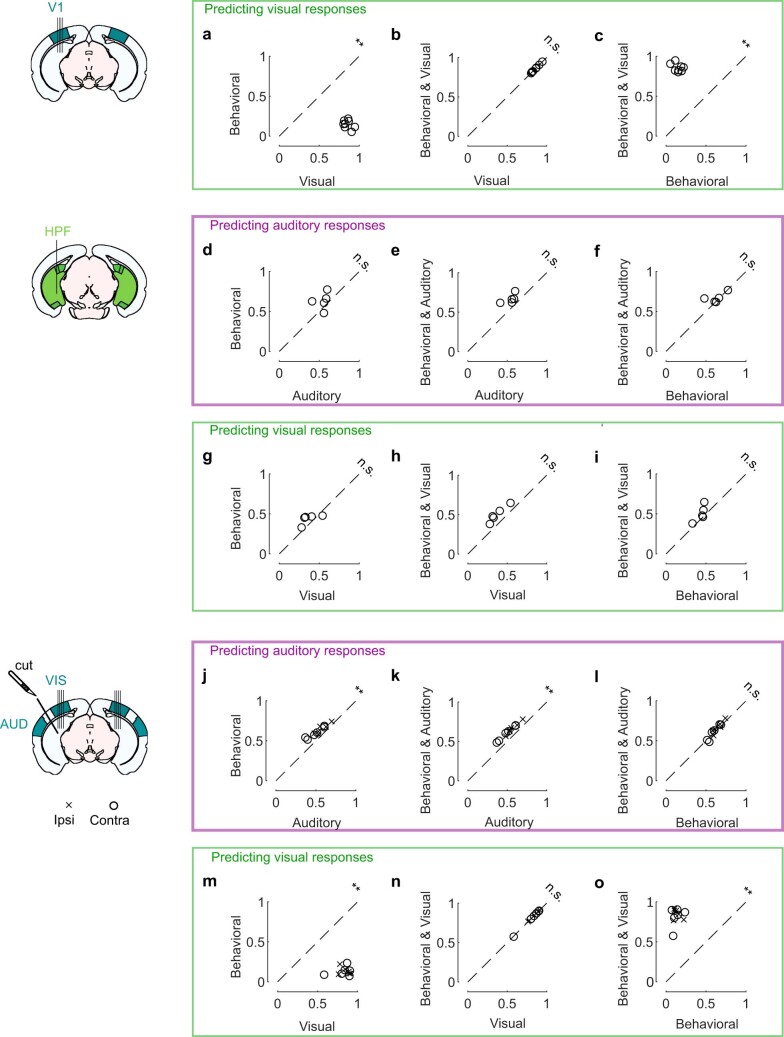
Movements predict activity evoked by sounds in visual cortex and HPF, and by videos in HPF. **a-c**. Cross-validated correlation of the visual responses and their predictions for all mice, comparing 3 different models: one with videos only (‘Visual’), one with eye and body movements only (‘Behavioral’), and one with all predictors (‘Full’) (**: p = 0.0078, n = 8 mice). **d-f**. Same as **a-c** but for auditory responses for the HPF recordings (albeit the low number of animals did not allow for conclusions on significance). (n = 5 mice) **g-i**. Same as **a-c** but for visual responses for the HPF recordings. **j-l**. Same as **a-c** but for auditory responses for the transectomy experiment recordings (**: p = 0.00049, n = 12 recordings across 3 mice). **m-o**. Same as **a-c** but for visual responses for the transectomy experiment recordings. All tests are two-sided paired Wilcoxon sign rank test.

By contrast, and indeed as expected for a brain region that encodes images, a purely visual model explained a large fraction of the activity evoked in V1 by videos while the behavioral model did not (Extended Data Figs. 1g and 8a–c,j–o). Behavior explained a much smaller fraction, mainly along visual PC1, which does not dominate the visual responses the way auditory PC1 dominates the auditory responses. In the hippocampal formation, finally, the behavioral model explained both the sound- and video-evoked activity, suggesting that any visual or auditory activity observed there is largely related to movements (Extended Data Figs. 1g and 8d–i).

**Extended Data Fig. 9:**
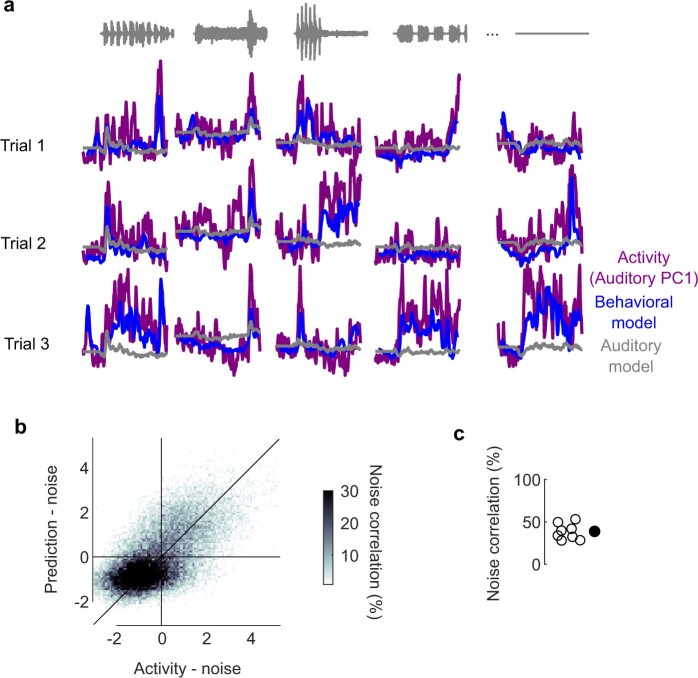
Sound-evoked body movements and sound-evoked brain activity fluctuate together. **a**. Single-trial, sound-related activity along auditory PC1 for one example mouse (*purple*). The prediction from the auditory model (gray) and the behavioral model (*blue*) are shown. **b**. Correlation between the single-trial noise in neural activity along auditory PC1 and the single-trial noise in the prediction for the same example mouse. **c**. Correlation values for all mice (open dots) and their average (filled dot).

Further confirming the role of body movements, we found that trial-by-trial variations in sound-evoked V1 activity were well-predicted by trial-by-trial variations in body movement (Extended Data Fig. 9). The movements elicited by each sound were stereotyped but not identical across trials. The behavioral model and the full model captured these trial-by-trial variations, which could not be captured by the auditory model because (by definition) the sounds did not vary across repeats. The trial-by-trial variations of V1’s auditory PC1 showed a correlation of 0.39 with its cross-validated prediction from movements (*P* = 0.0078, Wilcoxon two-sided signed rank test). In other words, the V1 activity evoked by sounds in individual trials followed a similar time course as the body movements observed in those trials.

**Extended Data Fig. 10:**
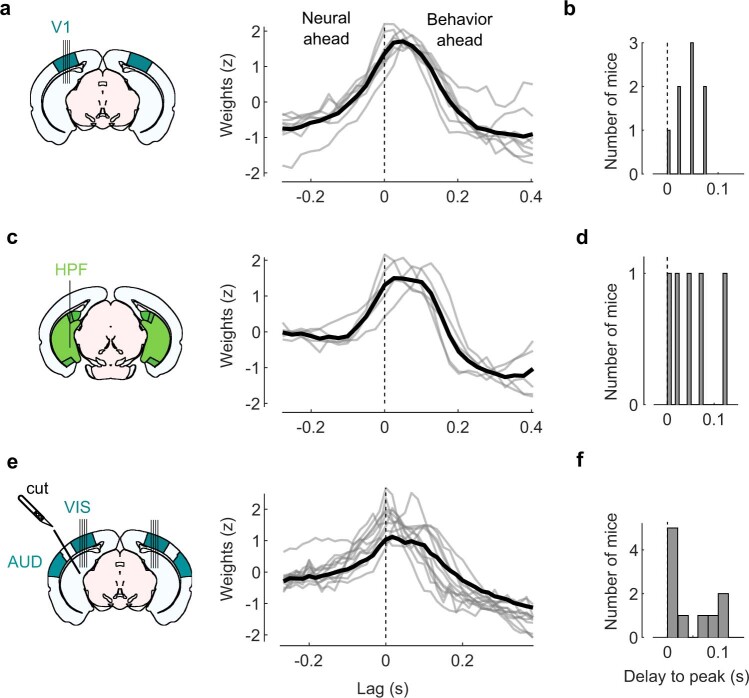
Body movements precede brain activity. **a**. Weights of the regression model to predict neural auditory PC1 from motion PCs (z-scored motion PC1 weights only are shown) for each individual mice (*gray*) and the average across mice (*black*). The model was computed on the spontaneous (no stimulus) period for the visual cortex experiments (Fig. 1). **b**. Distribution of the delay to the peak of the weights. A positive delay means that movement precedes and predicts neural activity by such a delay. **c**, **d**. Same as **a**, **b**, but for recordings in the HPF (Fig. 2). **e**,**f**. Same as **a**, **b**, but for recordings in visual cortex during the transectomy experiment (Fig. 3) (both sides).

Finally, the behavioral model confirmed the intuition obtained from the correlations (Fig. 4h)—movements preceded the activity evoked by sounds in visual cortex. The kernel of a behavioral model fit to predict auditory PC1 during spontaneous activity showed that movement could best predict neural activity occurring 25–50 ms later (Extended Data Fig. 10). This suggests that the activity evoked by sounds in visual cortex is driven by changes in internal and behavioral state.

## Discussion

These results confirm that sounds evoke activity in visual cortex^2–7^ but provide an alternative interpretation for this activity based on the widespread neural correlates of internal state and body movement^10,12–14,27^. We found that different sounds evoke different uninstructed body movements such as whisking, which reflect rapid changes in the internal state. Crucially, we discovered that these movements are sufficient to explain the activity evoked by sounds in visual cortex in our experiments. These results suggest that, at least in our experiments, the sound-evoked activity had a behavioral origin.

Confirming this interpretation, we found that sound-evoked activity in visual cortex was independent of projections from auditory cortex. This result contrasts those of studies that ascribed the activity evoked by sounds in V1 to a direct input from auditory cortex. These studies used multiple methods: silencing of auditory cortex^2,5^, stimulation of its projections to visual cortex^2,3^ or transectomy of these projections^2^. However, the first two methods would interfere with auditory processing, and thus could affect sound-evoked behavior. We thus opted for transectomy^2^, which is less likely to modify behavior, and we performed bilateral recordings to have an internal control—the uncut side—within the same mice and with the same behavior. In accordance with our interpretation, these manipulations did not reduce sound-evoked activity in V1.

This result contrasts with the original study that introduced the transectomy^2^, and the difference in results may be due to differences in methods. First, the previous study was conducted intracellularly and mostly in layers 2/3 (where sounds hyperpolarized cells, unlike in other layers where sounds increased spiking), whereas we recorded extracellularly mainly in layers 4-6 (and observed mainly increases in spiking). Second, the previous study performed recordings hours after the transectomy, whereas we performed them days later. Third, the previous study anesthetized the mice, whereas we did not, a difference that can profoundly affect V1 activity^28^.

Our results indicate that sound-evoked activity is widespread in visual cortex and even in the hippocampal formation, and in both regions, it is low-dimensional. These properties echo those of movement-related activity, which is distributed all over the brain^13,14,22,27^ and low-dimensional^13^. We indeed found that movement-related neural activity even in the absence of sounds spanned essentially the same dimensions as sound-evoked activity. Moreover, the movements elicited by the sounds in each trial accurately predicted the subsequent sound-evoked activity. This is remarkable considering that all the movements we measured are in the face, and that our analyses are linear. It is possible that movements of other body parts, or more complex analyses, would provide even better predictions of the neural activity elicited by sounds.

Our findings do not exclude the possibility of genuine auditory signals inherited by visual cortex from auditory cortex. After all, projections from auditory to visual cortex exist and may carry auditory signals for other stimuli or in other behavioral contexts. Moreover, some discrepancies between our results and the literature^2–7^ could be due to differences in recording techniques and in the associated sampling biases^29^. Our V1 recordings were biased toward layers 4–6. However, layer 2/3 also exhibits substantial movement-related activity^10–14^, so its sound-evoked activity might be similarly explained by movements. Finally, it is also possible that auditory projections affect only a minor fraction of V1 neurons, or that they affect neurons that do not fire at high rates, and that we missed these neurons in our recordings.

Distinguishing putative auditory signals from the large contribution of internal state and behavior will require careful and systematic controls, which are rarely performed in passively listening mice. Some studies have controlled for eye movements^5^ or for overt behaviors such as licking^7^. However, even these studies may have overlooked the types of movement that we observed to correlate with neuronal activity, which were subtle twitches of the whiskers or the snout (Supplementary Video 1). An exception is a study^20^ that explored the contribution of whisking to sound-evoked activity V1 neurons in layer 1. In agreement with our results, this study found that whisking explains a fraction of those neurons’ sound-evoked activity. However, it did not explain all the neural activity. This discrepancy could be due to differences in recording methods (two-photon imaging versus electrophysiology) in cortical layers (layer 1 versus layers 4–6) or in the analyses. For instance, the previous study relied on a hard threshold to call a response auditory versus movement-related, whereas we estimated the fraction of sound-evoked activity explained by movement.

Our results do not imply that cortical activity is directly due to body movements; instead, cortical activity and body movements may both arise from changes in the internal state. Consistent with this view, we found that sound-evoked activity in V1 is low-dimensional, and thus different from the high-dimensional representation of visual stimuli^13^. This interpretation would explain some of the sound-evoked activity in visual cortex under anesthesia^2,3^, where movements are not possible, but state changes are common and difficult to control and monitor^30,31^.

Finally, these observations suggest that changes in states or behavior may also explain other aspects of neural activity that have been previously interpreted as being multisensory^9^. Stereotyped body movements can be elicited not only by sounds^16–19^ but also by images^32–36^ and odors^33,37^. For instance, in our experiments, the videos evoked visual responses in both V1 and in hippocampal formation, and the latter could be largely explained by video-evoked body movements. Such movements may be even more likely in response to natural stimuli^19^, which are increasingly common in the field. Given the extensive correlates of body movement observed throughout the brain^13,14,21,27,38^, these observations reinforce the importance of monitoring behavioral state and body movement when interpreting sensory-evoked activity.

## Methods

Experimental procedures at UCL were conducted according to the UK Animals Scientific Procedures Act (1986), approved by the Animal Welfare and Ethical Review Body (AWERB) at UCL and performed under personal and project licenses released by the Home Office following appropriate ethics review.

### Surgery and recordings

Recordings were performed on eight mice (six male and two female), between 16 and 38 weeks of age. Mice were first implanted with a headplate designed for head fixation under isoflurane anesthesia (1–3% in O_2_). After recovery, neural activity was recorded using Neuropixels 1.0 (*n* = 5) and 2.0 (*n* = 3, among which two had four shanks) probes implanted in left primary visual cortex (2.5 mm lateral, 3.5 mm posterior from Bregma, one probe per animal) and in the underlying hippocampal formation. In five of the mice, the probes were implanted permanently or with a recoverable implant as described in refs. 24, 39, and in the remaining three, they were implanted with a recoverable implant of a different design (Yoh Isogai and Daniel Regester, personal communication). Results were not affected by the implantation strategy. Electrophysiology data were acquired using SpikeGLX (https://billkarsh.github.io/SpikeGLX/, versions 20190413, 20190919 and 20201012). Sessions were automatically spike-sorted using Kilosort2 (https://github.com/MouseLand/Kilosort/releases/tag/v2.0 (ref. 40)) and manually curated to select isolated single cells using Phy (https://github.com/cortex-lab/phy). Because spike contamination is a key source of bias^29^, we took particular care in selecting cells with few or no violations in interspike interval (ISI), and we confirmed that a key measure used in our study, the reliability of auditory responses, did not correlate with the ISI violations score. In fact, it showed a slightly negative correlation, indicating that the best-isolated neurons tended to have the highest reliability. Reliability for both auditory and visual responses also grew with firing rate, as may be expected. The final number of cells was 640 in the primary visual cortex (eight mice, 69/53/54/44/31/33/144/212 for each recording) and 233 in the hippocampal formation (five mice, 49/15/28/64/77 for each recording, mainly from dorsal subiculum and prosubiculum). Probe location was checked post hoc by aligning it to the Allen Mouse Brain Atlas^41^ visually or through custom software (www.github.com/petersaj/AP_histology).

Before and in between experiments, mice were housed in individually ventilated cages (IVC), with a 9 am light/9 pm dark cycle (no reverse/shifted light cycle). Temperature was maintained between 20 °C and 24 °C and humidity was maintained between 50 and 70%.

### Transectomy experiments

In three additional mice (all male of 10, 21 and 22 weeks of age), we performed transectomies to cut the fibers running from auditory to visual cortex and followed them with bilateral recordings in visual cortex. Mice expressed GCaMP6s in excitatory neurons (mouse 1 and 3: Rorb.Camk2tTA.Ai96G6s_L_001; mouse 2: tet0-G6s x CaMK-tTA) so we could monitor the activity of the intact visual cortex through widefield imaging (data not shown). Before headplate implantation, we used a dental drill (13,000 rpm) to perform a narrow rectangular (0.3-mm wide) craniotomy along the anteroposterior axis (from 1.6 mm posterior to 4.3 mm posterior) centered at 4.3 mm lateral to Bregma. To make the transectomy, we then used an angled micro knife (angled 15°, 10315-12 from Fine Science Tools), mounted on a Leica digital stereotaxic manipulator with a fine drive. Ensuring the skull was in a horizontal position (the difference between both DV coordinates did not exceed 0.1 mm), the knife was tilted 40° relative to the brain. The knife was inserted to a depth of 1.7 mm at the posterior end of the craniotomy and slowly moved to the anterior end with the manipulator control. Any bleeding was stemmed by applying gelfoam soaked in cortex buffer. To protect the brain, we then applied a layer of Kwik-Sil (World Precision Instruments) followed by a generous layer of optical adhesive (NOA 81, Norland Products). Following this, we attached a headplate to the skull as described above and covered any exposed parts of the skull with more optical adhesive.

After a rest period of 1 week for recovery, we imaged the visual cortex under a widefield scope to confirm that it was healthy and responding normally to visual stimuli. Bilateral craniotomies were performed between 7 d and 14 d following the transectomy, and acute bilateral recordings were acquired using 4-shank Neuropixels 2.0 probes targeting visual cortex over multiple days (3, 1 and 2 consecutive days in the three mice). The total number of cells was 1059 (ipsi) and 914 (contra) (per recording, ipsi/contra: 164/185; 216/106; 254/324; 58/59; 218/125; 149/115). We imaged the brains using serial section^42^ two-photon^43^ tomography. Our microscope was controlled by ScanImage Basic (Vidrio Technologies) using BakingTray (https://github.com/SainsburyWellcomeCentre/BakingTray, 10.5281/zenodo.3631609). Images were assembled using StitchIt (https://github.com/SainsburyWellcomeCentre/StitchIt, https://zenodo.org/badge/latestdoi/57851444). Probe location was checked using brainreg^44–46^, showing that most recordings were in area V1, and partially VISpm and VISl. The exact location of the probe in visual cortex did not affect the results so we pooled all areas together under the name of visual.

### Stimuli

In each session, mice were presented with a sequence of audio, visual or audiovisual movies, using Rigbox (https://github.com/cortex-lab/Rigbox, version 2.3.1). The stimuli consisted of all combinations of auditory and visual streams extracted from a set of 11 naturalistic movies depicting the movement of animals such as cats, donkeys and seals, from the AudioSet database^25^. An additional visual stream consisted of a static full-field gray image and an additional auditory stream contained no sound. Movies lasted for 4 s and were separated by an intertrial interval of 2 s. The same randomized sequence of movies was repeated four times during each experiment, with the first and second repeat separated by a 5-min interval.

The movies were gray-scaled, spatially rescaled to match the dimensions of a single screen of the display, and duplicated across the three screens. The visual stream was sampled at 30 frames per second. Visual stimuli were presented through three displays (Adafruit, LP097QX1), each with a resolution of 1024 × 768 pixels. The screens covered approximately 270 × 70 degrees of visual angle, with 0 degrees being directly in front of the mouse. The screens had a refresh rate of 60 frames per second and were fitted with Fresnel lenses (Wuxi Bohai Optics, BHPA220-2-5) to ensure approximately equal luminance across viewing angles.

Sounds were presented through a pair of Logitech Z313 speakers placed below the screens. The auditory stream was sampled at 44.1 kHz with two channels and was scaled to a sound level of −20 dB relative to full scale.

In situ sound intensity and spectral content were estimated using a calibrated microphone (GRAS 40BF 1/4’ Ext. Polarized Free-field Microphone) positioned where the mice sit, and reference loudness was estimated using an acoustic calibrator (SV 30A; Supplementary Fig. 1). Mice were systematically habituated to the rig through 3 d of familiarizing with the rig’s environment and head-fixation sessions of progressive duration (from 10 min to 1 h). They were not habituated to the specific stimuli before the experiment. Two exceptions were the transectomy experiments, where mice were presented with the same protocol across the consecutive days of recordings (so a recording on day 2 would mean the mouse had been through the protocol once already), and in specific control experiments not shown here (*n* = 2 mice). Presentation of the sounds over days (from 2 to 5 d) did not alter the observed behavioral and neural responses (*n* = 2 transectomy mice + 2 control mice).

### Videography

Eye and body movements were monitored by illuminating the subject with infrared light (830 nm, Mightex SLS-0208-A). The right eye was monitored with a camera (The Imaging Source, DMK 23U618) fitted with zoom lens (Thorlabs MVL7000) and long-pass filter (Thorlabs FEL0750), recording at 100 Hz. Body movements (face, ears, front paws and part of the back) were monitored with another camera (same model but with a different lens, Thorlabs MVL16M23) situated above the central screen, recording at 40 Hz for the experiments in V1 and HPF (Figs. 1 and 2) and 60 Hz for the transectomy experiments (Fig. 3). Video and stimulus time were aligned using the strobe pulses generated by the cameras, recorded alongside the output of a screen-monitoring photodiode and the input to the speakers, all sampled at 2,500 Hz. Video data was acquired on the computer using mmmGUI (https://github.com/cortex-lab/mmmGUI). To compute the singular value decompositions of the face movie and to fit pupil area and position, we used the facemap algorithm^13^ (www.github.com/MouseLand/facemap, MATLAB version).

### Behavior-only experiments

To test for the influence of basic acoustic properties on movements, we ran behavior-only experiments (that is, only with cameras filming the mice, and no electrophysiology; Supplementary Fig. 2) on eight mice in which we played (1) white noise of various intensities, (2) pure tones of various frequencies and (3) white noise coming from various locations. In contrast with the previous experiments, auditory stimuli were presented using an array of seven speakers (102-1299-ND, Digikey), arranged below the screens at 30° azimuthal intervals from −60° to +60° (where −90°/+90° is directly to the left/right of the subject). Speakers were driven with an internal sound card (STRIX SOAR, ASUS) and a custom seven-channel amplifier (http://maxhunter.me/portfolio/7champ/). As in the previous experiments, in situ sound intensity and spectral content were estimated using a calibrated microphone (GRAS 40BF 1/4ʼ Ext. Polarized Free-field Microphone) positioned where the mice sit, and reference loudness was estimated using an acoustic calibrator (SV 30A). Body movements were monitored with a Chameleon3 camera (CM3-U3-13Y3C-S-BD, Teledyne FLIR) recording at 60 Hz. The movie was then processed with facemap.

The effect of each factor was then quantified using repeated-measures ANOVA with either the sound loudness, frequency or location as a factor.

### Data processing

MATLAB 2019b and 2022a were used for data analysis. For each experiment, the neural responses constitute a five-dimensional array *D* of size *N*_*t*_ time bins × *N*_*v*_ videos × *N*_*a*_ sounds × *N*_*r*_ repeats × *N*_*c*_ cells. The elements of this matrix are the responses *d*_*tvarc*_ measured at time *i*, in video *v*, sound *a*, repeat *r* and cell *c*. *D* contains the binned firing rates (30 ms bin size) around the stimulus onset (from 1 s before onset to 3.8 s after onset), smoothed with a causal half Gaussian filter (standard deviation of 43 ms), and *z* scored for each neuron.

Pupil area and eye position were baseline-corrected to remove the slow fluctuations and focus on the fast, stimulus-evoked and trial-based fluctuations—the mean value of the pupil area or eye position over the second preceding stimulus onset was subtracted from each trial. Signed eye motion (horizontal and vertical) was computed as the difference of the eye position between time bins. The unsigned motion was obtained as the absolute value of the signed motion. The global eye motion was estimated as the absolute value of the movement in any direction (L2 norm). Eye variables values during identified blinks were interpolated based on their values before and after the identified blink. Body motion variables were defined as the first 128 body motion PCs. Both eye-related and body-related variables were then binned similarly to the neural data. We note that the timing precision for the face motion is limited by both the camera acquisition frame rate (40 fps, not aligned to stimulus onset), and the binning used here (30-ms bins, aligned on stimulus onset). Thus, real timings can differ by up to 25 ms.

All analyses that needed cross-validation (test–retest component covariance, decoding and prediction) were performed using a training set consisting of half of the trials (odd trials) and a test set based on the other half (even trials). Models were computed on the train set and tested on the test set. Then test and train sets were swapped, and quantities of interest were averaged over the two folds.

To estimate the correlation of the sound-evoked time courses across mice, the variable of interest was split between training and test set, averaged over all trials (for example, for sound-related activity, over videos and repeats), and the Pearson correlation coefficient was computed between the training set activity for each mouse and the test set activity of all mice (thus giving a cross-validated estimate of the auto- and the cross-correlation). Averages were obtained by Fisher’s Z-transforming each coefficient, averaging, and back-transforming this average.

### Marginalization

To isolate the contribution of videos or sounds in the neural activity, we used a marginalization procedure similar to the one used in factorial ANOVA. By *d*_*tvarc*_, we denote the firing rate of cell *c* to repeat *r* of the combination of auditory stimulus *a* and visual stimulus *v*, at time *t* after stimulus onset. The marginalization procedure decomposes *d*_*tvarc*_ into components that are equal across stimuli, related to videos only, related to sounds only, related to audiovisual interactions and noise:

$$d_{tvarc} = m_{tc} + v_{tvc} + a_{tac} + i_{tvac} + {\it{\epsilon }}_{tvarc}$$

The first term is the mean of the population activity across videos, sounds and repeats:

$$m_{tc} = d_{t \cdot\cdot\cdot c} = \frac{1}{{N_vN_aN_r}}\mathop {\sum }\limits_v \mathop {\sum }\limits_a \mathop {\sum }\limits_r d_{tvarc}$$

where dots in the second term indicate averages over the missing subscripts, and *N*_*v*_, *N*_*a*_, *N*_*r*_ denote the total number of visual stimuli, auditory stimuli and repeats.

The second term, the video-related component, is the average of the population responses over sounds and repeats, relative to this mean response:

$$\begin{array}{*{20}{c}} {v_{tvc} = d_{tv \cdot\cdot c} - m_{tc}} \end{array}$$

Similarly, the sound-related component is the average over videos and repeats, relative to the mean response:

$$\begin{array}{*{20}{c}} {a_{tac} = d_{t \cdot a \cdot c} - m_{tc}} \end{array}$$

The audiovisual interaction component is the variation in population responses that is specific to each pair of sound and video:

$$\begin{array}{*{20}{c}} {i_{tvac} = d_{tva \cdot c} - m_{tc} - v_{tvc} - a_{tac}} \end{array}$$

Finally, the noise component is the variation across trials:

$$\begin{array}{*{20}{c}} {\it{\epsilon }} \end{array}_{tvarc} = d_{tvarc} - d_{tva \cdot c}$$

In matrix notation, we will call *A*, *V* and *I* the arrays with elements *a*_*tac*_, *v*_*tvc*_ and *i*_*tvac*_ and size *N*_*t*_ × *N*_*a*_ × *N*_*c*_, *N*_*t*_ × *N*_*v*_ × *N*_*c*_, and *N*_*t*_ × *N*_*v*_ × *N*_*a*_ × *N*_*c*_.

### Dimensionality reduction

The arrays of sound-related activity *A*, of video-related activity *V* and of audiovisual interactions *I* describe the activity of many neurons. To summarize this activity, we used cvPCA^15^. In this approach, principal component projections are found from one-half of the data, and an unbiased estimate of the reliable signal variance is found by computing their covariance with the same projections on the second half of the data.

We illustrate this procedure on the sound-related activity. In what follows, all arrays, array elements and averages (for example, *A*, *a*_*tac*_, *a*_*t*·*c*_) refer to training-set data (odd-numbered repeats), unless explicitly indicated with the subscript test (for example, *A*_test_, *a*_*tac*;test_, *a*_*t*·*c*;test_).

We first isolate the sound-related activity *A* as described above from training set data (odd-numbered trials). We reshape this array to have two dimensions *N*_*t*_*N*_*a*_ × *N*_*c*_ and perform PCA: $T=AW$ where *T* (*N*_*t*_*N*_*a*_ × *N*_*p*_) is a set of time courses of the top *N*_*p*_ principal components of *A*, and *W* is the PCA weight matrix (*N*_*c*_ × *N*_*p*_).

For cvPCA analysis, we took *N*_*p*_ = *N*_*c*_ to estimate the amount of reliable stimulus-triggered variance in each dimension (Fig. 2f,i and Supplementary Fig. 2). We computed the projections of the mean response over a test set of even-numbered trials, using the same weight matrix: *T*_test_ = *A*_test_*W* and evaluated their covariance with the training-set projections:

$$\begin{array}{*{20}{c}} {\hat V_k = \frac{1}{{N_tN_a - 1}}\mathop {\sum }\limits_{j = 1}^{N_tN_a} \left( {t_{jk} - t_{ \cdot k}} \right)\left( {t_{jk;\mathrm{test}} - t_{ \cdot k;\mathrm{test}}} \right)} \end{array}$$

This method provides an unbiased estimate of the stimulus-related variance of each component^15^. Analogous methods were used to obtain the signal variance for principal components of the visual response and interaction, by replacing *A* with *V* or *I* (Supplementary Fig. 2). The cvPCA variances were normalized either by the sum for all auditory dimensions (for example, Fig. 2h j) or the sum for all dimensions from video-related, sound-related and interaction-related decompositions (Extended Data Fig. 2).

To determine if a cvPCA dimension had variance significantly above 0, we used a shuffling method. The shuffling was done by changing the labels of both the videos and the sounds for each repeat. We performed this randomization 1,000 times and chose a component to be significant if its test–retest covariance value was above the 99th percentile of the shuffled distribution. We defined the dimensionality as the number of significant components. For the video-related activity, we found an average of 79 significant components (±23 s.e., *n* = 8 mice). As expected, this number grew with the number of recorded neurons^15^ (data not shown). For the sound-related activity, instead, we found only four significant components on average (±1 s.e., *n* = 8 mice). For the interactions between videos and sounds, finally, we found zero significant components (0 ± 0 s.e., *n* = 8 mice) indicating that the population responses did not reflect significant interactions between videos and sounds.

For visualization of PC time courses (Figs. 1–3 and Extended Data Fig. 4), we computed the weight matrices *W* from the training set but we used the projection of the full dataset to compute the time courses of the first component. In Extended Data Fig. 1, instead, we computed *W* on the full dataset but we projected only the test set, to show the model’s cross-validated prediction.

### Decoding

Single-trial decoding for video- or sound identity was performed using a template-matching decoder applied to neural or behavioral data. In this description, we will focus on decoding sound identity from neural data. The data were again split into training and test sets consisting of odd and even trials. Both test and trained trials contained a balanced number of trials for each sound.

When decoding sound-related neural activity (Figs. 1–3 and Extended Data Fig. 1), we took *N*_*p*_ = 4, so the matrix *T* containing PC projections of the mean training-set sound-related activity had size *N*_*t*_*N*_*a*_ × 4; using more components did not affect the results. To decode the auditory stimulus presented on a given test-set trial, we first removed the video-related component by subtracting the mean response to the video presented on that trial (averaged over all training-set trials). We then projected this using the training-set weight matrix *W* to obtain a *N*_*t*_ × 4 time course for the top auditory PCs and found the best-matching auditory stimulus by comparing it to the mean training-set time courses for each auditory stimulus using Euclidean distance. A similar analysis was used to decode visual stimuli, using *N*_*p*_ = 30 components in visual cortex and *N*_*p*_ = 4 in the hippocampal formation.

To decode the sound identity from behavioral data, we used the *z*-scored eye variables (pupil area and eye motion in Extended Data Fig. 5) or the first 128 principal components of the motion energy of the face movie (Fig. 4) and performed the template-matching the same way as the with the neural data.

The significance of the decoding accuracy (compared to chance) was computed by performing a Wilcoxon right-sided signed rank test to compare to chance level (1/12), treating each mouse as independent. The comparison between video identity and sound identity decoding accuracy was computed by performing a paired Wilcoxon two-sided signed rank test across mice.

### Encoding

To predict neural activity from stimuli/behavioral variables (‘encoding model’; Fig. 4 and Supplementary Fig. 3), we again started by extracting audio- or video-related components and performing Principal Component Analysis, as described above. However, this time the weight matrices were computed from the full dataset rather than only the training set. Again, we illustrate by describing how sound-related activity was predicted, for which we kept *N*_*p*_ = 4 components; video-related activity was predicted similarly but with *N*_*p*_ = 30 in visual cortex and *N*_*p*_ = 4 in the hippocampal formation.

We predicted neural activity using linear regression. The target matrix *Y* contained the marginalized, sound-related activity on each trial, projected onto the top four auditory components: specifically, we compute the matrix with elements *d*_*tvarc*_− *m*_*tc*_− *v*_*tvc*_, reshape to a matrix of size *N*_*t*_*N*_*v*_*N*_*a*_*N*_*r*_ × *N*_*c*_, and multiply by the matrix of PC weights *W*. We predicted *Y* by regression: *Y* ≈ *XB*, where *X* is a feature matrix and *B* are weights fit by cross-validated ridge regression.

The feature matrix depended on the model. To predict from sensory stimulus identity (see ‘auditory predictors’ in Supplementary Fig. 3), *X* had one column for each combination of auditory stimulus and peristimulus timepoint, making *N*_*a*_*N*_*t*_ = 1,524 columns, *N*_*t*_*N*_*v*_*N*_*a*_*N*_*r*_ rows, and contained 1 during stimulus presentations in a column reflecting the stimulus identity and peristimulus time. With this feature matrix, the weights *B* represent the mean activity time course for each dimension and stimulus, and estimation is equivalent to averaging across the repeats of the train set. It is thus equivalent to a test–retest estimation and is not a model based on acoustic features of the sounds.

To predict from behavior, we used features for pupil area, pupil position (horizontal and vertical), eye motion (horizontal and vertical signed and unsigned), global eye motion (L2 norm of *x* and *y* motion, unsigned), blinks (thus nine eye-related predictors) and the first 128 face motion PCs, with lags from −100 to 200 ms (thus 12 lags per predictor, 1,644 predictors total, see ‘eye predictors’ and ‘body motion predictors’ in Supplementary Fig. 3). As for the neural activity target matrix *Y*, all behavioral variables were first marginalized to extract the sound-related modulations. To predict from both stimulus identity and behavior, we concatenated the feature matrices, obtaining a matrix with 3,168 columns. The beginning and end of the time course for each trial were padded with NaNs (12—the number of lags—at the beginning and end of each trial, to avoid cross-trial predictions by temporal filters. Thus, the feature matrix has (*N*_*t*_ + 24)*N*_*v*_*N*_*a*_*N*_*r*_ rows. A model with the eye variables only and a model with the face motion variables only were also constructed (Extended Data Fig. 7). Note that in the case of mice for which the eye was not recorded (two of the eight mice, and all transectomy experiments), the behavioral model contained only the body motion variables.

We used ridge regression to predict the single-trial version of *Y* from *X* on the training set. The best lambda parameter was selected using a threefold cross-validation within the training set.

To measure the accuracy of predicting trial-averaged sound-related activity (Fig. 4), we averaged the *N*_*t*_*N*_*v*_*N*_*a*_*N*_*r*_ /2 × *N*_*p*_ activity matrix *Y*_test_ over all test-set trials of a given auditory stimulus, to obtain a matrix of size *N*_*t*_*N*_*a*_ × *N*_*c*_, and did the same for the prediction matrix *X*_test_*B*, and evaluated prediction quality by the elementwise Pearson correlation of these two matrices.

To evaluate predictions of trial-to-trial fluctuations (Extended Data Fig. 9b,c), we computed a ‘noise’ matrix of size *N*_*t*_*N*_*v*_*N*_*a*_*N*_*r*_ /2 × *N*_*p*_ by subtracting the mean response to each sound: $y_{tvarp;\,\mathrm{test}} - y_{t \cdot a \cdot p;\,\mathrm{test}}$, performed the same subtraction on the prediction matrix *X*_test_*B*, and evaluated prediction quality by the elementwise Pearson correlation of these two matrices. Again, the average was obtained by Fisher’s Z-transforming each coefficient, averaging, and back-transforming this average.

To visualize the facial areas important to explain neural activity (Extended Data Fig. 7), we reconstructed the weights of the auditory PC1 prediction in pixel space. Let ${{{\mathbf{b}}}}_0^{{{{\mathrm{body}}}}}$ (1 × 128) be the weights predicting neural auditory PC1 at lag 0 from each of the 128 body motion PCs. Let *ω* (128 × total number of pixels in the video) be the weights of each of these 128 face motion PCs in pixel space (as an output of the facemap algorithm). We obtained an image *i* of the pixel-to-neural weights by computing ${{{I}}} = {{{\mathbf{b}}}}_0^{{{{\mathrm{body}}}}}{{{\omega }}}$.

Finally, to explore the timing relationship between movement and neural activity, we looked at the cross-correlogram of the motion PC1 and the auditory PC1 during the spontaneous (no stimulus) period (Fig. 4 and Extended Data Fig. 6). The auditory PC1 was found by computing its weights without cross-validation. To maximize the temporal resolution, the regression analysis was performed on the spikes sampled at the rate of the camera acquisition (40 fps, thus 25 ms precision). We then computed the lag associated with the cross-correlogram, which showed that movement preceded neural activity by 25–50 ms. To avoid errors induced by ‘large’ cross-correlograms due to the autocorrelation of the two signals, we also performed a ridge regression of the auditory PC1 from the motion PCs during the spontaneous period and looked at the peak of the weights of motion PC1 to predict auditory PC1 (Extended Data Fig. 10).

### Movement- and sound-related subspaces overlap

To quantify the overlap between the movement- and the sound-related subspaces of neural activity in V1, we computed how much of the sound-related variance the movement-related subspace could explain^13^. We first computed the movement-related subspace by computing a reduced-rank regression model to predict the neural activity matrix *S* (*N*_*s*_ × *N*_*c*_, with *N*_*s*_ being the number of time points) from the motion components matrix *M* with lags (*N*_*s*_ × 2,688, with the latter dimension coming from 128 motion components × 21 lags) during the spontaneous period (no stimulus), both binned at the face video frame rate (40 or 60 Hz). This yields a weight matrix *B* (2,688 × *N*_*c*_) so that: *S* *≈* *MB*. The weight matrix *B* factorizes as a product of two matrices of sizes 2,688 × *r* and *r* × *N*_*c*_, with *r* being the rank of the reduced-rank regression. The second part of this factorization, the matrix of size *r* × *N*_*c*_ of which transpose we call *C* (*N*_*c*_ × *r*), forms an orthonormal basis of the movement-related subspace of dimensionality *r*. Here, we chose *r* = 4 to match the size of the sound-related subspace, but the results were not affected by small changes in this value. Then, we projected the sound-related activity of the train set *A* and the test set *A*_test_ onto *C* and measured the covariance of these projections for each dimension of the movement-related subspace. This is similar to the cvPCA performed above to find the variance explained by auditory PCs, except the components are here the ones most explained by behavior and not by sound. The overlap between the movement-related and the sound-related subspaces was finally quantified as the ratio of the sound-related variance explained by the first four components of each subspace.

We note that the fact that the overlap between the sound-related subspace and the behavior-related subspace is not 100% may come from the noise in estimating the behavior-related subspace, which relies on the spontaneous period only which was less than 25 min.

### Transectomy quantification

To visualize and estimate the extent of the transectomy, we used the software brainreg^44–46^ (https://github.com/brainglobe/brainreg) to register the brain to the Allen Mouse Brain Reference Atlas^41^ and manually trace the contours of the cut using brainreg-segment (https://github.com/brainglobe/brainreg-segment). The cut was identified visually by observing the massive neuronal loss (made obvious by a loss of fluorescence) and scars.

To estimate the extent of the fibers that were cut by the transectomy, we took advantage of the large-scale connectivity database of experiments performed by the Allen Brain Institute (Allen Mouse Brain Connectivity Atlas^26^, https://connectivity.brain-map.org/). Using custom Python scripts, we selected and downloaded the 53 experiments where injections were performed in the auditory cortex and projections were observed in visual cortex (we subselected areas V1, VISualpm and VISl as targets because these were where the recordings were performed). We used the fiber tractography data to get the fibers’ coordinates in the reference space of the Allen Mouse Brain Atlas, to which was also aligned the actual brain and the cut reconstruction. Using custom software, we selected only the fibers of which terminal were inside or within 50 µm of either ipsilateral or contralateral visual cortex. We identified the cut fibers as all fibers that were passing inside or within 50 µm of the cut. Because auditory cortex on one side sends projections to both sides (yet much more to the ipsilateral side), cutting the fibers on one side could also affect responses on the other side. Moreover, residual sound-evoked activity on the side ipsilateral to the transectomy could possibly be explained by fibers coming from the contralateral auditory cortex. We thus quantified the auditory input to each visual cortex as the number of intact fibers coming from both auditory cortices, with one side being cut and the other being intact. We then made the hypothesis that the size of the responses, or more generally the variance explained by sounds in both populations, would linearly reflect these ‘auditory inputs’. We then compared the sound-related variance on the cut side to its prediction from the sound-related variance on the uncut side. This provided an internal control, with the same sounds and behavior. We took the sound-related variance as the cumulative sum of the variance explained by the first four auditory PCs, on both sides. We then used brainrender^47^ (https://github.com/brainglobe/brainrender/releases/tag/v2.0.0.0) to visualize all results.

### Reporting summary

Further information on research design is available in the Nature Portfolio Reporting Summary linked to this article.

## Online content

Any methods, additional references, Nature Portfolio reporting summaries, source data, extended data, supplementary information, acknowledgements, peer review information; details of author contributions and competing interests; and statements of data and code availability are available at 10.1038/s41593-022-01227-x.

## Supplementary information

Supplementary Information Supplementary Figs. 1–3.

Reporting Summary

Supplementary Video 1 Sounds evoked stereotyped movements and brain responses. Left—time courses of the sound amplitude, neural auditory PC1, face motion PC1 and pupil size, for 4 different trials (colors). Right—visual stimuli, view of the mouse’s face, motion energy and view of the mouse’s eye for the same trials (left to right).

## Data Availability

Preprocessed data can be accessed at 10.6084/m9.figshare.21371247.v2. Raw data are available from the authors upon reasonable request. Stimuli were selected from the AudioSet Database (https://research.google.com/audioset/). Connectivity patterns between auditory and visual cortices were extracted from the Allen Mouse Brain Connectivity Atlas (https://connectivity.brain-map.org/), and the exact list of experiments selected can be accessed at https://github.com/cbimbo/Bimbard2022/blob/main/transecAnat/projection_search_results.csv.

## Code availability

Code for data analysis can be accessed at https://github.com/cbimbo/Bimbard2022 (10.5281/zenodo.7253394).
