# Supplementary material for: Behavioral origin of sound-evoked activity in mouse visual cortex
Source: Nat Neurosci. Author manuscript; Available in PMC 2023 Feb 9. (PMC9905016; doi:10.1038/s41593-022-01227-x)
Supplement: Supplementary Data [file EMS156467-supplement-Supplementary_Data.pdf]

Supplementary information

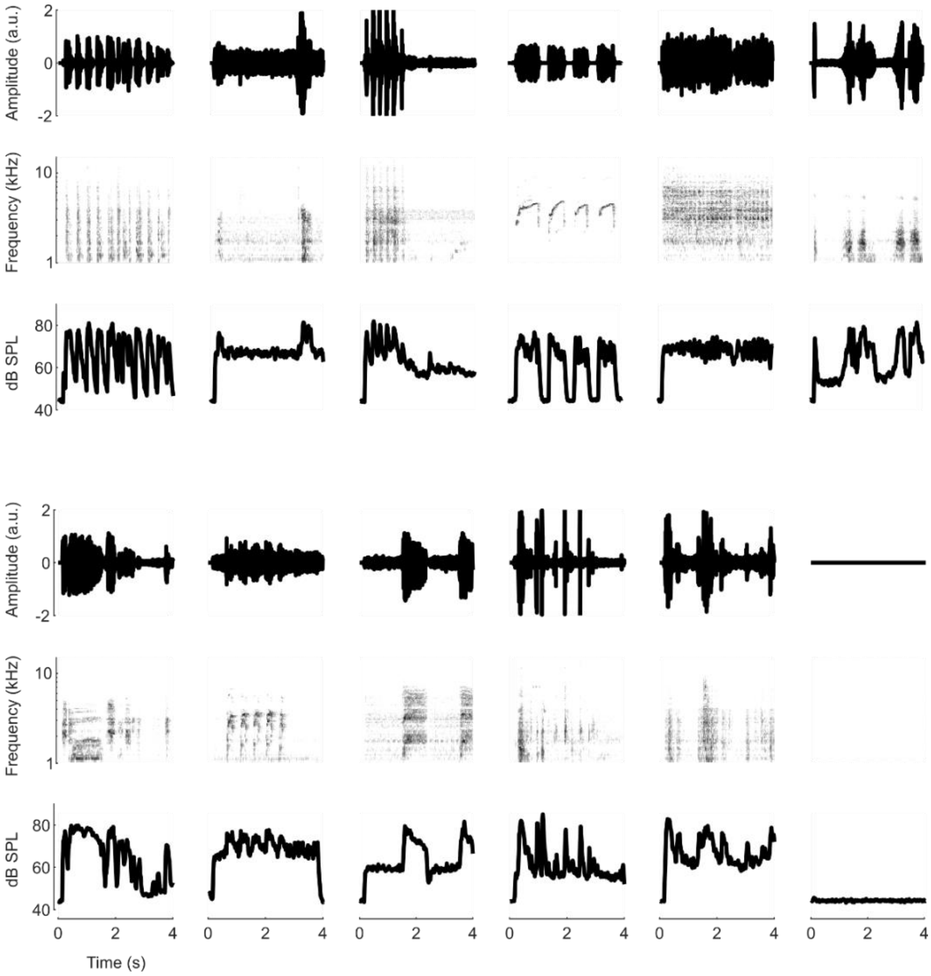

Supplementary Fig. 1. **Naturalistic sounds used in this study: spectral content and loudness.** For each sound is displayed: top: amplitude; middle: frequency spectrum; bottom: loudness.

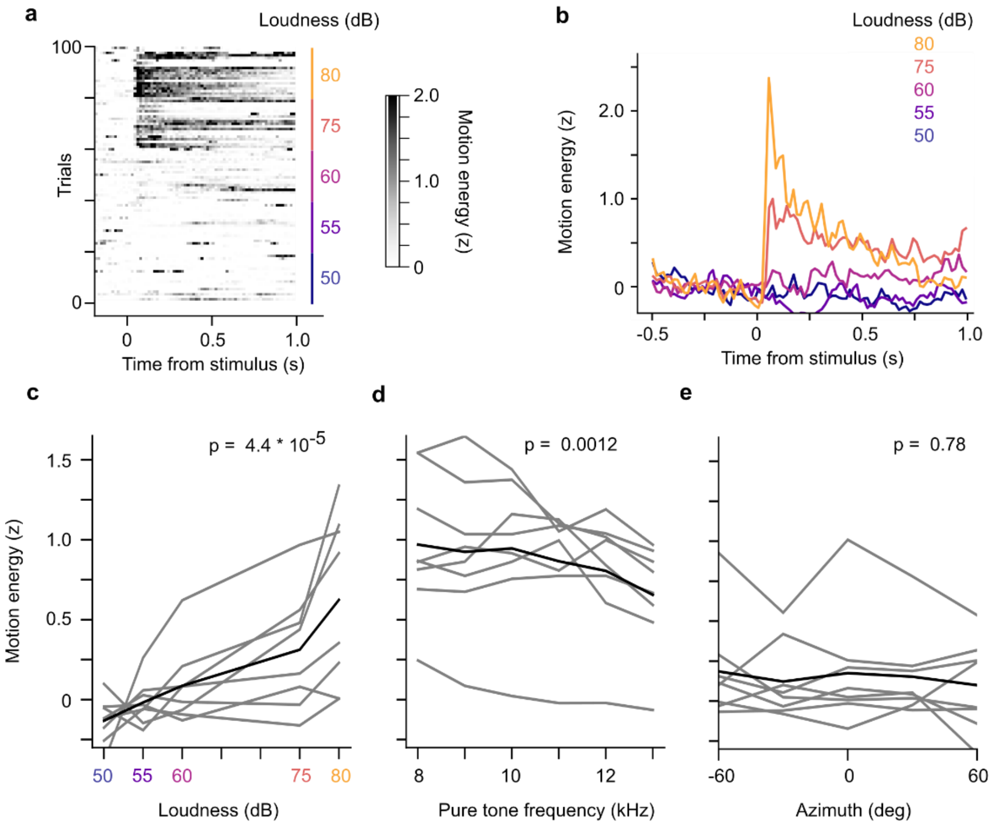

Supplementary Fig. 2. **Loudness is the main driver of uninstructed behavioral responses. a.** Raster of the average motion of an example mouse in response to white noise bursts of different loudness (from 50 to 80 dB SPL). **b.** Peri-stimulus time histograms of the average motion for the same example mouse for different sound volumes. **c.** Average motion energy of 6 different mice as a function of loudness. **d**. Same as **c**, but for a pure tone of different frequencies (60 dB). **e**. Same as **c**, but for a white noise burst played from different azimuthal locations (80 dB). P-values were computed using repeated-measures ANOVA with either the sound loudness, frequency, or location as a factor.

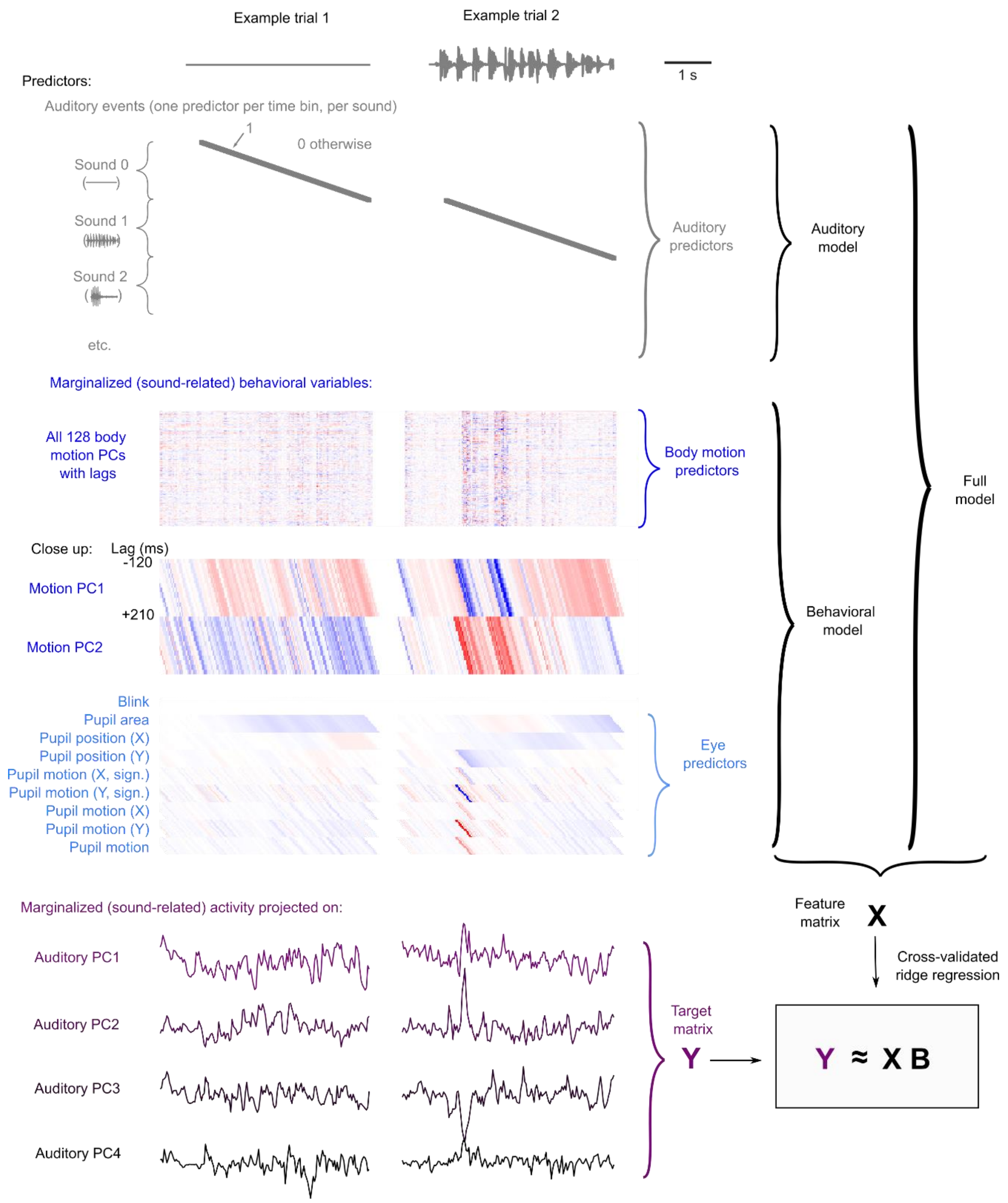

Supplementary Fig. 3. **Structure of the models.** The feature matrix **X** depends on the model. In the auditory model, there are as many predictors as the number of sounds multiplied by the number of time bins during which the sound is played (grey dots show where the value is 1, and 0 otherwise). In the behavioral model, the predictors consist of all 128 motions PCs, with various eye variables (see Methods), with 12 different lags (from -120 to +210ms). A close up for the first 2 motion PCs allows for a better visualization of the predictors with different lags. The target matrix **Y** is composed of the projections of the marginalized activity (sound responses only) onto the sound-related subspace (first 4 auditory PCs). Finally, the full model combines both the auditory and the behavioral predictors. The model is then fitted using 3-fold cross-validated ridge regression.
