# Supplementary material for: Behavioral origin of sound-evoked activity in mouse visual cortex
Source: Nat Neurosci. 2023 Jan 9;26(2):251–8. doi: 10.1038/s41593-022-01227-x (PMC9905016; doi:10.1038/s41593-022-01227-x)
Supplement: Supplementary file 1 [file 41593_2022_1227_MOESM1_ESM.pdf]

nature neuroscience

Article

https://doi.org/10.1038/s41593-022-01227-x

Behavioral origin of sound-evoked activity in mouse visual cortex

In the format provided by the authors and unedited

Supplementary information

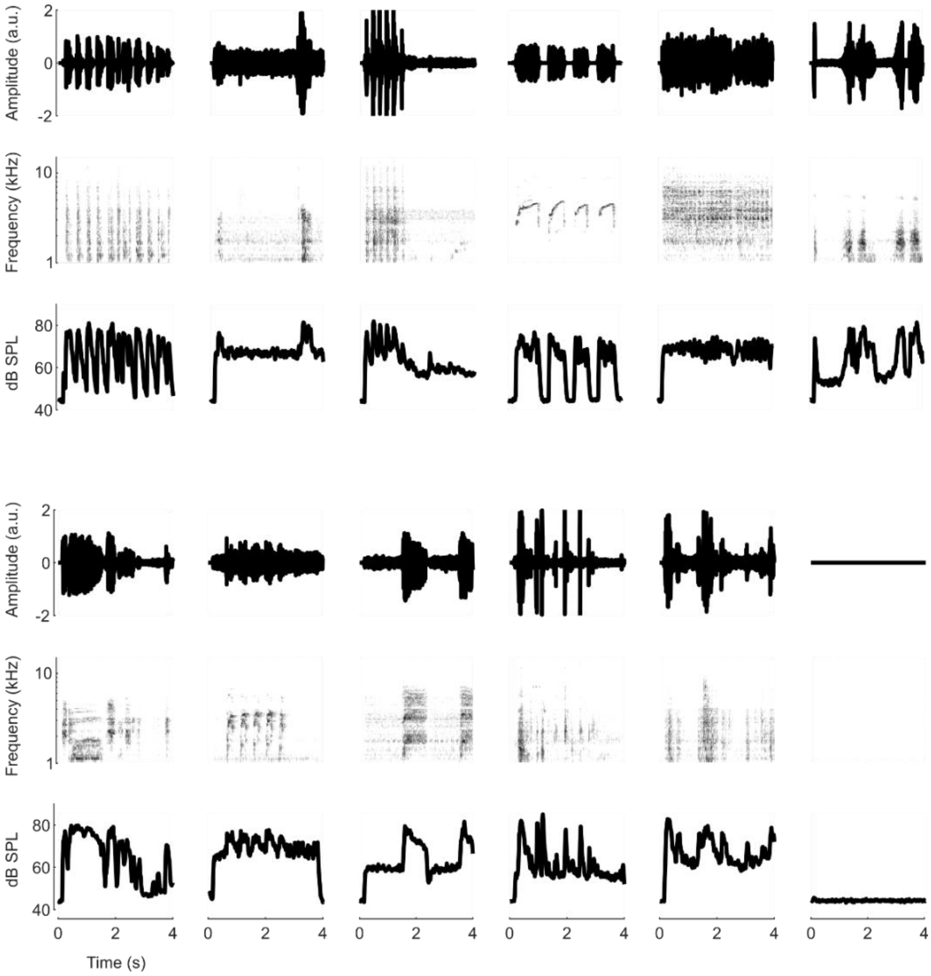

Supplementary Fig. 1. **Naturalistic sounds used in this study: spectral content and loudness.** For each of the 12 sounds, each column shows the sound amplitude (*top*), frequency spectrum (*middle*) and loudness (*bottom*).

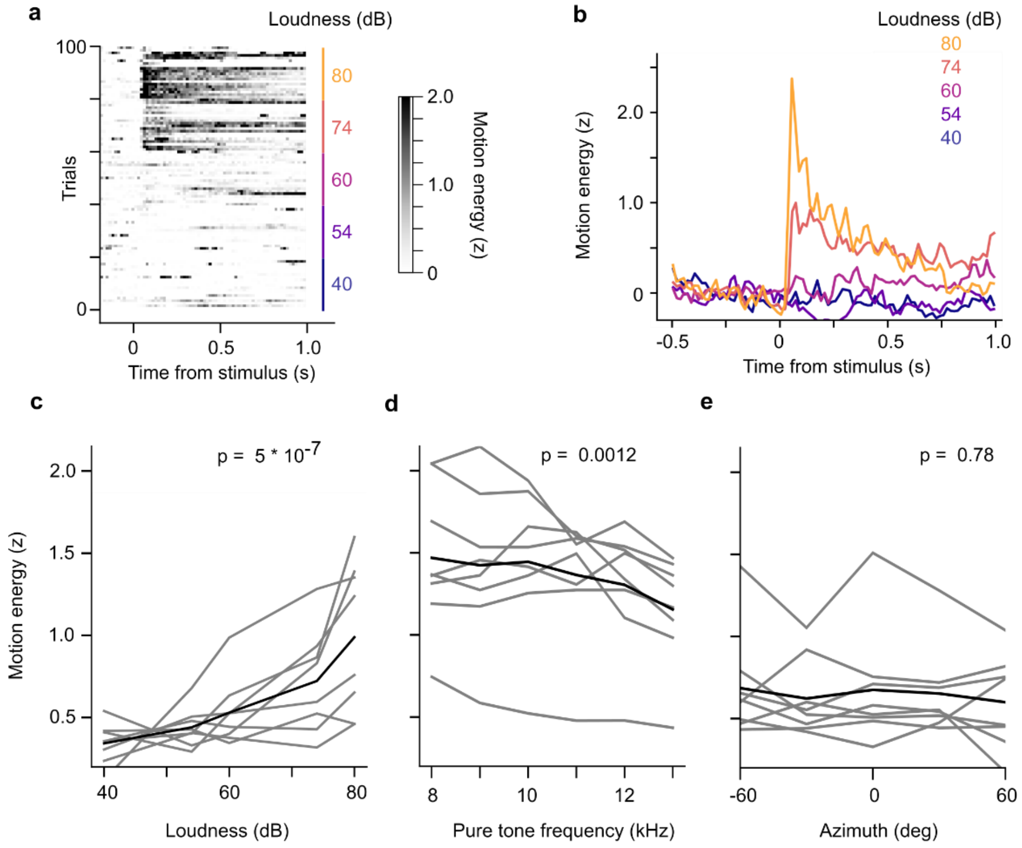

Supplementary Fig. 2. **Loudness is the main driver of uninstructed behavioral responses. a.** Raster of the average motion of an example mouse in response to white noise bursts of different loudness (from 40 to 80 dB SPL). **b.** Peri-stimulus time histograms of the average motion for the same example mouse for different sound volumes. **c.** Average motion energy of 6 different mice as a function of loudness. **d**. Same as **c**, but for a pure tone of different frequencies (60 dB). **e**. Same as **c**, but for a white noise burst played from different azimuthal locations (80 dB). P-values were computed using repeated-measures ANOVA with either the sound loudness, frequency, or location as a factor.

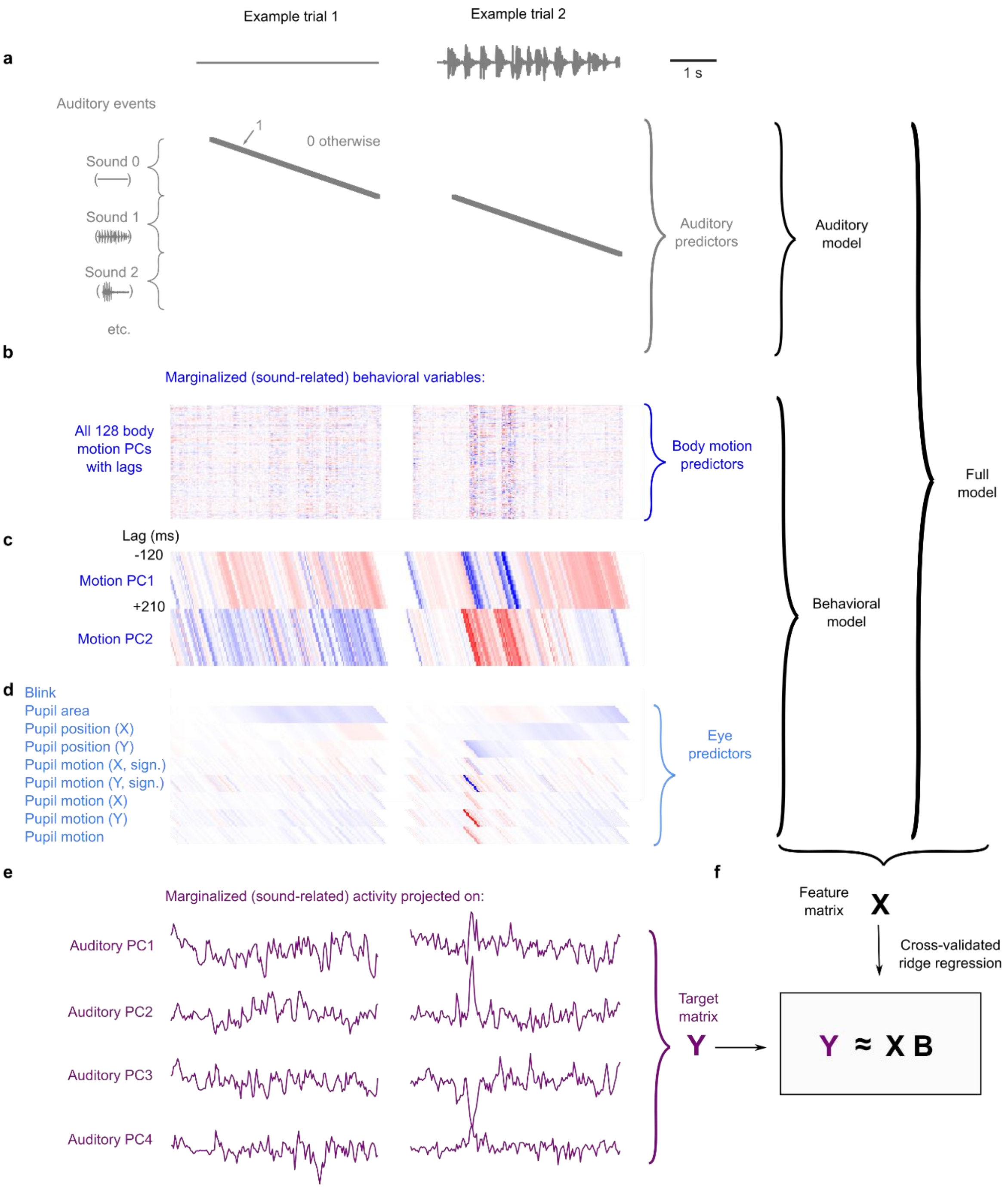

Supplementary Fig. 3. **Structure of the models. a.** The feature matrix for the auditory model involves only the auditory predictors. It is 1 in the time bins during which a sound is played (grey dots), and 0 otherwise. **b**. The feature matrix for the behavioral model includes all 128 motions PCs with 12 lags (from -120 to +210 ms). **c**. A closeup for the first 2 motion PCs allows for a better visualization of the predictors with different lags. **d**. Same as **b**, but for various eye variables. The same lags are used. **e.** The target matrix **Y** is composed of the projections of the marginalized activity (sound responses only) onto the sound-related subspace (first 4 auditory PCs). **f.** The feature matrix **X** depends on the model: auditory, behavioral, or full model (which combines both the auditory and the behavioral predictors). It is fitted to the target matrix **Y** using 3-fold cross-validated ridge regression to obtain the matrix of regression weights **B**.
