# Supplementary material for: Behavioral origin of sound-evoked activity in mouse visual cortex
Source: Nat Neurosci. 2023 Jan 9;26(2):251–8. doi: 10.1038/s41593-022-01227-x (PMC9905016; doi:10.1038/s41593-022-01227-x)
Supplement: Supplementary file 2 [file 41593_2022_1227_MOESM2_ESM.pdf]

nature portfolio

Corresponding author(s): Celian Bimbard

Last updated by author(s): Oct 21, 2022

Reporting Summary

Nature Portfolio wishes to improve the reproducibility of the work that we publish. This form provides structure for consistency and transparency in reporting. For further information on Nature Portfolio policies, see our Editorial Policies and the Editorial Policy Checklist.

Statistics

For all statistical analyses, confirm that the following items are present in the figure legend, table legend, main text, or Methods section.

n/a	Confirmed	
☐	☒	The exact sample size (*n*) for each experimental group/condition, given as a discrete number and unit of measurement
☐	☒	A statement on whether measurements were taken from distinct samples or whether the same sample was measured repeatedly
☐	☒	The statistical test(s) used AND whether they are one- or two-sided<br>*Only common tests should be described solely by name; describe more complex techniques in the Methods section.*
☐	☒	A description of all covariates tested
☒	☐	A description of any assumptions or corrections, such as tests of normality and adjustment for multiple comparisons
☐	☒	A full description of the statistical parameters including central tendency (e.g. means) or other basic estimates (e.g. regression coefficient) AND variation (e.g. standard deviation) or associated estimates of uncertainty (e.g. confidence intervals)
☐	☒	For null hypothesis testing, the test statistic (e.g. *F*, *t*, *r*) with confidence intervals, effect sizes, degrees of freedom and *P* value noted<br>*Give P values as exact values whenever suitable.*
☒	☐	For Bayesian analysis, information on the choice of priors and Markov chain Monte Carlo settings
☒	☐	For hierarchical and complex designs, identification of the appropriate level for tests and full reporting of outcomes
☐	☒	Estimates of effect sizes (e.g. Cohen's *d*, Pearson's *r*), indicating how they were calculated

Our web collection on statistics for biologists contains articles on many of the points above.

Software and code

Policy information about availability of computer code

Data collection	Rigbox (https://github.com/cortex-lab/Rigbox, version 2.3.1)<br>SpikeGLX (https://billkarsh.github.io/SpikeGLX/, versions 20190413, 20190919, and 20201012)<br>mmmGUI (https://github.com/cortex-lab/mmmGUI)
Data analysis	MATLAB 2019b and 2022a<br>Kilosort2 (https://github.com/MouseLand/Kilosort/releases/tag/v2.0)<br>Phy (https://github.com/cortex-lab/phy)<br>Facemap (www.github.com/MouseLand/facemap, MATLAB version)<br>BakingTray (https://github.com/SainsburyWellcomeCentre/BakingTray, https://doi.org/10.5281/zenodo.3631609)<br>Stitchit (https://github.com/SainsburyWellcomeCentre/StitchIt, https://zenodo.org/badge/latestdoi/57851444)<br>Histology alignment tool (www.github.com/petersaj/AP_histology)<br>Brainreg 0.1.5 (https://github.com/brainglobe/brainreg)<br>Brainreg-segment (https://github.com/brainglobe/brainreg-segment)<br>Brainrender v2.0.0.0 (https://github.com/brainglobe/brainrender/releases/tag/v2.0.0.0)<br>Custom code created for this research (https://github.com/cbimbo/Bimbard2022, https://doi.org/10.5281/zenodo.7253394)

For manuscripts utilizing custom algorithms or software that are central to the research but not yet described in published literature, software must be made available to editors and reviewers. We strongly encourage code deposition in a community repository (e.g. GitHub). See the Nature Portfolio guidelines for submitting code & software for further information.

Data

Policy information about availability of data

All manuscripts must include a data availability statement. This statement should provide the following information, where applicable:

- Accession codes, unique identifiers, or web links for publicly available datasets
- A description of any restrictions on data availability
- For clinical datasets or third party data, please ensure that the statement adheres to our policy

Preprocessed data can be accessed at https://doi.org/10.6084/m9.figshare.21371247.v2. Raw data are too large (hundreds of GBs) to be shared on a publicly available repository, but will be available from the authors upon reasonable request.
Stimuli were selected from the AudioSet Database (https://research.google.com/audioset/). Connectivity patterns between auditory and visual cortices were extracted from the Allen Mouse Brain Connectivity Atlas (https://connectivity.brain-map.org/), and the exact list of experiments selected can be accessed at https://github.com/cbimbo/Bimbard2022/blob/main/transecAnat/projection_search_results.csv.

Human research participants

Policy information about studies involving human research participants and Sex and Gender in Research.

Reporting on sex and gender	N/A
Population characteristics	N/A
Recruitment	N/A
Ethics oversight	N/A

Note that full information on the approval of the study protocol must also be provided in the manuscript.

Field-specific reporting

Please select the one below that is the best fit for your research. If you are not sure, read the appropriate sections before making your selection.

☒ Life sciences ☐ Behavioural & social sciences ☐ Ecological, evolutionary & environmental sciences

For a reference copy of the document with all sections, see nature.com/documents/nr-reporting-summary-flat.pdf

Life sciences study design

All studies must disclose on these points even when the disclosure is negative.

Sample size	We used n=8 mice for our initial experiment and n=3 mice in the transectomy experiment, that further confirmed our results. The number of mice was chosen based on the expected effect sizes from previous studies (Iurilli et al., 2012, Ibrahim et al., 2016) and on the current standard used for mice in neuroscience for such effect sizes and sufficient statistical power.
Data exclusions	No data was excluded from this study.
Replication	Similar results were obtained across mice, and in two independent sets of experiments (n=8 and n=3 mice), with all analysis replicated in all animals.
Randomization	Randomization was used for stimulus presentation. No comparison across groups was performed, and controls were performed within mice for the area comparisons (VIS vs. HPF) and the transectomy experiment.
Blinding	No blinding was used in this study. Blinding was not necessary because no comparison across groups was made, and the main control were performed simultaneously in the same mice (comparison across areas recorded simultaneously, and bilateral recordings for transectomy experiment).

Reporting for specific materials, systems and methods

We require information from authors about some types of materials, experimental systems and methods used in many studies. Here, indicate whether each material, system or method listed is relevant to your study. If you are not sure if a list item applies to your research, read the appropriate section before selecting a response.

Materials & experimental systems

n/a	Involved in the study	
☒	☐	Antibodies
☒	☐	Eukaryotic cell lines
☒	☐	Palaeontology and archaeology
☐	☒	Animals and other organisms
☒	☐	Clinical data
☒	☐	Dual use research of concern

Methods

n/a	Involved in the study	
☒	☐	ChIP-seq
☒	☐	Flow cytometry
☒	☐	MRI-based neuroimaging

Animals and other research organisms

Policy information about studies involving animals; ARRIVE guidelines recommended for reporting animal research, and Sex and Gender in Research

Laboratory animals	8 mice (6 male and 2 female), between 16 and 38 weeks of age, C57/bl6.<br>Additional 3 mice, all male, of 10, 21 and 22 weeks of age (2 mice Rorb.Camk2tTA.Ai96G6s_L_001 and 1 mouse: tet0-G6s x CaMK-tTA).<br>Mice were housed in IVC (individually ventilated cages), with a 9am-9pm light/dark cycle (no reverse/shifted light cycle), temperature was maintained between 20-24 degree celcius and humidity was maintained between 50-70%.
Wild animals	This study did not involve wild animals.
Reporting on sex	Our results held across sexes (9 male, 2 female). No further analysis on sex differences were performed.
Field-collected samples	This study did not involve field-collected samples.
Ethics oversight	Experimental procedures at UCL were conducted according to the UK Animals Scientific Procedures Act (1986), approved by the Animal Welfare and Ethical Review Body (AWERB) at UCL and under personal and project licenses released by the Home Office following appropriate ethics review.

Note that full information on the approval of the study protocol must also be provided in the manuscript.
